# Advances in Bioslurry Remediation of Potentially Toxic Elements (PTEs) in Polluted Soils

**DOI:** 10.3390/jox16060187

**Published:** 2026-10-01

**Authors:** Nadine Othman, Claudio Cocozza, Giorgio S. Senesi, Danilo Vona, Carmine Crecchio, Jalal Halwani, Roberto Terzano

**Affiliations:** 1 Department of Soil, Plant and Food Sciences, University of Bari “Aldo Moro”, Via Amendola 165/A, 70126 Bari, Italy; nadine.othman@uniba.it (N.O.); claudio.cocozza@uniba.it (C.C.); danilo.vona@uniba.it (D.V.); carmine.crecchio@uniba.it (C.C.); 2 CNR—Istituto per la Scienza e Tecnologia dei Plasmi (ISTP) Sede di Bari, Via Amendola 122/O, 70126 Bari, Italy; giorgio.senesi@cnr.it; 3 Doctoral School of Science and Technology, Lebanese University, Tripoli P.O. Box 6573/14, Lebanon; jhalwani@ul.edu.lb

**Keywords:** bioreactors, bioremediation, microorganisms, bioleaching, siderophores, immobilized bacteria, analytical techniques

## Abstract

Soil contamination by potentially toxic elements (PTEs) remains a major global challenge due to their persistence, toxicity, and bioaccumulation capacity. Despite being widely used for the ex situ remediation of soils polluted by organic contaminants, bioslurry reactors (BSRs) have been scarcely employed for the bioremediation of PTE-polluted soils. BSRs are indeed capable of treating a wide range of contaminated soils, which are often unsuitable for remediation by conventional biological treatments. BSRs allow control of mixing, aeration, and nutrient conditions, thus enhancing mass transfer, pollutant desorption, and microbe–pollutant interactions. In addition, diverse microbial metabolic mechanisms can be exploited to mobilize or transform PTEs through bioleaching, redox reactions, complexation, and acid generation. Numerous studies have demonstrated substantial removal efficiencies for PTEs, such as Cd, Co, Cu, Ni, Zn, and As. Despite their advantages, large-scale implementation of BSRs faces challenges related to operational costs, reactor design, energy demand, and the need for precise control of physicochemical parameters. Advances in microorganism immobilization, genetic engineering, and online parameter monitoring may represent levers for a wider application of this technology. This review represents the first systematical attempt to address the issue of BSR application to PTE-polluted soil remediation. The principles of BSR functioning are discussed together with the mechanisms of microbial bioleaching, highlighting the potentialities of BSR technology and addressing gaps and opportunities to fully exploit BSRs as a sustainable and economically viable tool for remediating PTE-polluted soils.

## 1. Introduction

Soil contamination is a form of land degradation mainly caused by the increasing number and intensity of anthropogenic activities beyond a sustainable limit for the environment [1,2]. In particular, the whole globe is facing the issue of soil pollution caused by potentially toxic elements (PTEs) that has raised concerns for human health and food security. Over the last two decades, soil pollution by PTEs has emerged as a major driver of land degradation and biodiversity loss, with reported adverse effects reaching 94.7% in plants, 77.4% in humans, and 68.4% in animals [3].

The PTEs that are reported to cause serious adverse effects on organisms include Sb, As, Cd, Cr(VI), Co, Cu, Pb, Hg, Mo, Ni, Se, Ag, Sn, Tl, V, and Zn [2,4,5]. These elements, comprising metals, metalloids, and non-metals, are characterized by persistence, toxicity, bioaccumulation, and biomagnification at elevated concentrations, posing risks to ecosystems and human health [6]. Although some of them are also micronutrients for many living organisms, high concentrations may result in toxicity [7,8]. Soils serve as major sinks for these elements, which can ultimately enter the food chain through leaching into water sources, plant uptake, and animal transfer [4,9].

The remediation of contaminated soils is essential for restoring polluted sites, supporting sustainable land use, and ensuring responsible environmental management. Physical, chemical, and biological methods are commonly used, whose selection depends on remediation targets and risk management needs [3,10].

In situ technologies treat contaminated soils directly on site, while ex situ methods involve soil excavation for treatment in dedicated facilities [11]. These approaches are categorized into five types: physical, chemical, electrical, thermal, and biological; or grouped into three broader classes: containment-based (e.g., capping), transformation-based (e.g., stabilization), and transport-based (e.g., extraction). Each technology has distinct mechanisms, benefits, and limitations, as discussed in Section 2.

Beyond containment, remediation may aim at repairing degraded ecosystems by approaches including halting degradation, restoring original conditions, or rehabilitating ecosystem functions. In this sense, bioremediation is a particularly important option, as it uses plants and microorganisms to mobilize, accumulate, or stabilize PTEs, thus reducing or eliminating their toxicity [12].

Among bioremediation technologies exploiting microbial activity, bioleaching can be used to remediate PTE-polluted soils by promoting their mobilization from insoluble or scarcely soluble forms by biological dissolution through complexation mechanisms or bio-oxidation. In fact, microbial metabolism can promote soil acidification or secrete complexing molecules which can dissolve PTEs from insoluble minerals and favor PTE leaching in nutritious environments [13]. Such processes are usually carried out off-site in suitable bioreactors to control all the parameters and avoid undesired migration of mobilized pollutants.

Slurry-phase biological treatment or bioslurry reactors (BSRs)—also referred to as soil-slurry bioreactors—are in fact preferred when a rapid and safe remediation is required, especially under conditions unsuitable for the use of conventional biological treatments, such as dry or low-permeable soils and/or recalcitrant/highly toxic pollutants and/or multiple contamination. Such systems enhance gas/liquid and solid/liquid mass transfers, improving contaminant bioavailability; reduce toxic pollutant concentrations via dilution with water; and allow the precise control of critical remediation parameters [14].

Recent reviews dealing with bioslurry technology have predominantly focused on organic contaminants, including PAHs, PCBs, petroleum hydrocarbons, pesticides, and other refractory organics [15,16,17]. On the other hand, no specific reviews dedicated to the application of BSRs for the remediation of soils polluted by PTEs have been published in the last few years. Moreover, most existing studies on the subject have focused on the application of different types of microorganisms or on the effects of external operating conditions on PTE mobilization and removal, while less attention has been given to maintaining or improving microbial activity and stability within the reactor. These limitations can be overcome by bacterial immobilization, representing a promising strategy to improve microbial retention, activity, and process efficiency in reactor systems. Yet, the potential for increasing stability, reproducibility, and practical performance of BSR-based PTE remediation has not been sufficiently evaluated.

In this review paper, besides describing the basic principles of BSR technology and its application to PTE-contaminated soils, an overview of the biochemical mechanisms of PTE bioleaching is presented, with particular attention to microbial siderophores as promising natural lixiviants for PTE extraction from polluted soils. Furthermore, a special focus has been dedicated to the methods and materials for bacteria immobilization, discussing advantages and disadvantages from the viewpoint of large-scale practical applications. For the preparation of this paper, a literature review has been conducted across major international bibliographic databases, including Web of Science, Scopus, and PubMed/MEDLINE. The search encompassed studies published mainly from 2011 to 2026, allowing both seminal and recent research to be captured. The last database search was conducted in August 2026. To ensure methodological rigor and the inclusion of high-quality evidence, priority was given to articles published in Q1-ranked journals, together with highly cited review articles and authoritative academic publications, including books and book chapters. Literature references dating before 2011 were also included, being cited as basic reference literature in the consulted bibliography. The main keywords used for the search were “soil remediation”, “potentially toxic elements (PTE)” or “potentially toxic metals (PTM)” or “heavy metals (HM) or “metals”, “bioreactors”, “bioslurry”, and “slurry bioremediation”. Other keywords such as “bacteria immobilization” or “immobilized bacteria”, “enzyme immobilization”, “bioleaching”, and “siderophores” were used for other topics covered in the manuscript.

## 2. Soil Contamination by Potentially Toxic Elements (PTEs) and Remediation

Potentially toxic elements can be present in soil, either naturally, due to rocks weathering and/or atmospheric depositions, or as a result of anthropogenic activities [8,18,19]. In particular, the most impacting human practices releasing PTEs in soil include mining and smelting activities, disposal of PTE-containing wastes and sludges, fossil fuel combustion, military operations, electronic waste disposal, irrigation with wastewater, and fertilizer application [3,20,21].

Soil is a highly complex and fragile system, composed of a mixture of solid phases (both organic and inorganic), water, air, and biological components. PTEs in soils may adsorb to clay minerals and/or soil organic matter and, depending on the PTE and soil characteristics, can also be partially available in the soil solution, thus endangering the entire soil ecosystem [22].

The fate and movement of PTEs in soil are largely determined by their chemical form and speciation. Once introduced into soil, PTEs undergo several processes such as precipitation/dissolution, adsorption/desorption, ion exchange, complexation with organic matter (OM, either solid or dissolved in water), and biological uptake (either by plants or soil (micro)organisms) [23].

The bioavailable fraction of PTEs is a crucial indicator for risk assessment and is controlled by soil physicochemical and biological conditions as well as element properties. Under typical soil conditions (i.e., pH 4–9, Eh +0.3–+0.9 V), cationic PTEs are adsorbed onto clay minerals, organic matter, and oxides, or precipitated as (hydr)oxides. Therefore, soil composition plays an important role, i.e., clay- and OM-rich soils with negative surface charges effectively retain cations, while sandy soils with poor OM content and low cation exchange capacity (CEC) promote their leaching. Furthermore, Fe and Al oxides, which are common in soils, can also bind PTEs in stable compounds, thus reducing their mobility and plant availability.

In general, a reduced cationic availability results from a higher content of negatively charged colloids (mainly OM, 2:1 phyllosilicates, and Fe/Mn oxides), and the formation of stable organometallic or insoluble inorganic compounds (e.g., with phosphates and carbonates). Thus, the identification of such soil factors is indispensable for proper environmental management. Among soil properties, pH plays a key role in influencing PTE mobility in soil; i.e., a low pH promotes PTEs mobilization while a high pH decreases the availability of cationic PTEs but increases that of anionic ones. For example, Cu and Pb sorption increases above pH 3–5, Co and Zn above 4–6, and Cd and Ni above 5–7 [24]. As pH declines, the increased activities of H^+^, Fe^3+^, Al^3+^, and their hydrolysed species enhance the competition with trace cationic metals for negatively charged sorption sites, thus promoting metal release into the soil liquid phase. Simultaneously, the protonation of mineral and organic surface functional groups reduces the net negative charge of soil colloids and may generate positive charges below the point of zero charge. Acidic conditions also destabilize key soil components, including Ca-carbonate and Al and Fe hydroxides, further increasing PTEs solubility. In particular, metal mobility below pH 6 typically increases in the order Cd > Zn > Ni > Mn > Cu > Pb > Hg. In contrast, elements that occur predominantly as anions, such as As, Mo, Se, and Cr (VI), are generally more mobile under alkaline conditions, as decreasing pH enhances anion sorption through the development of positive surface charges [25].

Over time, in situ and ex situ technologies have been developed to contain, clean, or restore soils contaminated by PTEs. Table 1 shows the mechanisms, advantages, disadvantages, and application status of the main available remediation techniques used for PTEs-contaminated soils.

**Figure 1:**
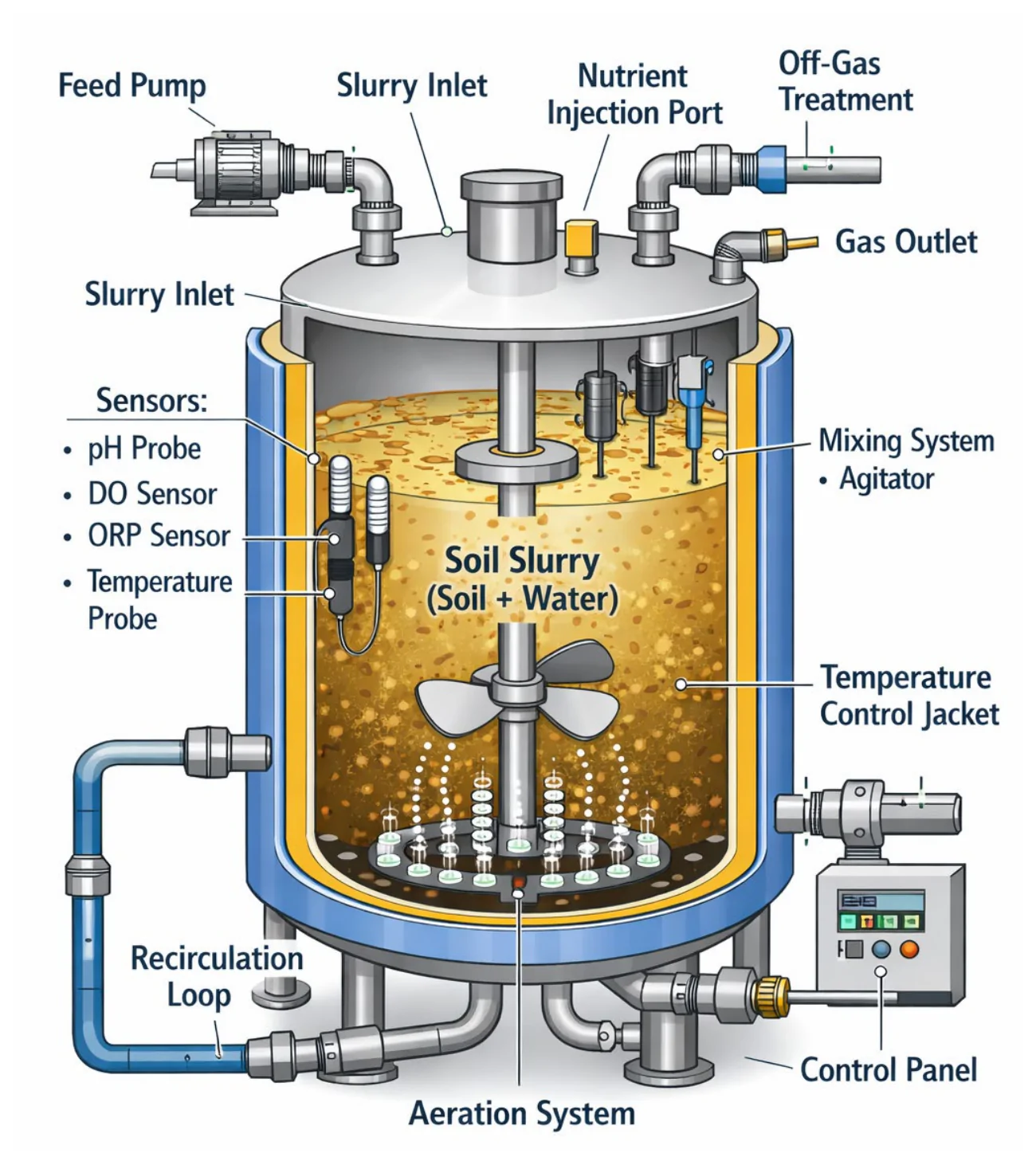
Scheme of a bioslurry tank reactor.

Among available technologies, the BSR involves the mixing of contaminated soil with water (10–40% *w*/*v*) and nutrients, followed by mechanical or pneumatic stirring to favor dispersion and stimulate microbial activity [35]. Bioslurry remediation integrates multiple bioremediation approaches [15,36] and differs from other bioremediation methods in that soil is fully suspended in water, thus facilitating homogeneous treatment [14,15]. The equipment typically includes soil pretreatment and conditioning units, a bioslurry reactor, a power supply, and auxiliary devices such as sedimentation, sewage, and gas treatment units (Figure 1). Operating modes can be batch, semi-continuous, or continuous, with batch systems being the most common; aerobic processes dominate, although anaerobic and mixed modes are emerging [15]. Aerobic BSRs include spargers or diffusers, while ancillary equipment ensures slurry conditioning and gas treatment [36].

A distinctive feature of BSRs is the treatment of soil in a near-homogeneous aqueous suspension and continuous mixing to enhance mass transfer, microbe–pollutant contact, and biodegradation rates, which result in shorter treatment times. BSRs also allow controlled use of different electron acceptors (O_2_, NO_3_^−^, SO_4_^2−^, CO_2_), environmental parameters optimization, and increased pollutant desorption via surfactants, complexing agents, or solvents [15,36].

In BSRs, the microbial community can be stimulated under controlled conditions to produce biomass and metabolites, including bioactive molecules that promote PTE leaching or stabilization processes [37].

The efficiency of contaminant removal relies not only on microbial activity but also on slurry rheology, hydrodynamics, gas–liquid oxygen transfer, and solid–liquid mass transfer. Pino-Herrera et al. [14] highlighted that contaminant removal in aerobic soil-slurry systems evolves from the interaction of biochemical mechanisms with gas–liquid and solid–liquid mass-transfer processes, and that reactor operating conditions and soil properties can highly impact these processes. In particular, the relatively high concentration of suspended soil particles differentiates BSRs from conventional aqueous bioreactors and makes the physical properties of the slurry a crucial factor to consider while designing a bioreactor. Thus, slurry rheology is specifically significant since increasing soil concentration can change viscosity, density, particle suspension, mixing, and, consequently, oxygen transfer. This effect is particularly relevant for fine-textured soils containing substantial clay fraction. Pino-Herrera et al. [38] experimentally investigated oxygen transfer in aerated and stirred clay slurries, finding that the presence of clay imposes a negative impact on the oxygen diffusion. The results of those authors indicated that, at higher clay concentrations, alterations in slurry density and viscosity are important contributors to the decrease in the oxygen-transfer coefficient, whereas bubble-related effects are more relevant at lower clay concentrations. These findings suggest that soil concentration should be considered not only as a biological operating parameter but also as a key hydrodynamic and mass-transfer factor in BSRs.

The relationship between slurry properties and oxygen transfer is particularly significant because oxygen availability can act as a limiting factor in aerobic soil bioremediation. Consequently, from an engineering perspective, mixing and aeration must be optimized simultaneously. Adequate stirring is essential to maintain soil particles in suspension and alleviate concentration gradients, while aeration must maintain sufficient oxygen to sustain aerobic microbial activity. However, increasing stirring and aeration also increases energy consumption, and increasing soil concentration can progressively enhance the resistance to mixing and oxygen transfer. Thus, the optimum operating conditions must be a compromise between attaining a sufficiently homogeneous slurry, maximizing mass transfer, and minimizing energy requirements.

At full scale, BSRs may involve large-lined lagoons or manufactured tanks ranging 3–25 m in diameter and 4.5–8 m in height, with capacities from 60 to 1000 m^3^ [36]. Recent market analysis estimates the global bioreactor market at approximately USD 1.2–1.3 billion in 2019, with a projected compound annual growth rate (CAGR) of about 6.8% for the period 2019–2025 [39]. This growth drives the development of more efficient, cost-effective BSRs through the optimization of design and operational factors such as flow rate, microbial concentration, temperature, pH, and stirring rate. Thus, implementing optimal parameters remains a central challenge for the soil remediation industry [37].

BSR technology, while offering significant environmental and practical benefits, does incur additional costs compared to some other bioremediation methods. These costs, however, remain economically advantageous when compared with many traditional physical remediation technologies. A comprehensive study by Sun et al. [15] emphasizes the necessity for in-depth research into the bioslurry remediation process to fully understand its economic and operational dynamics. The study also highlights that large-scale implementation of bioslurry technology faces several challenges, including technical complexities, scalability issues, and the need for specialized infrastructure. Thus, addressing these challenges is crucial for the broader adoption of bioslurry technology in environmental remediation efforts, especially for PTEs.

## 3. Slurry-Phase Bioreactors for PTE Soil Bioremediation

**Figure 2:**
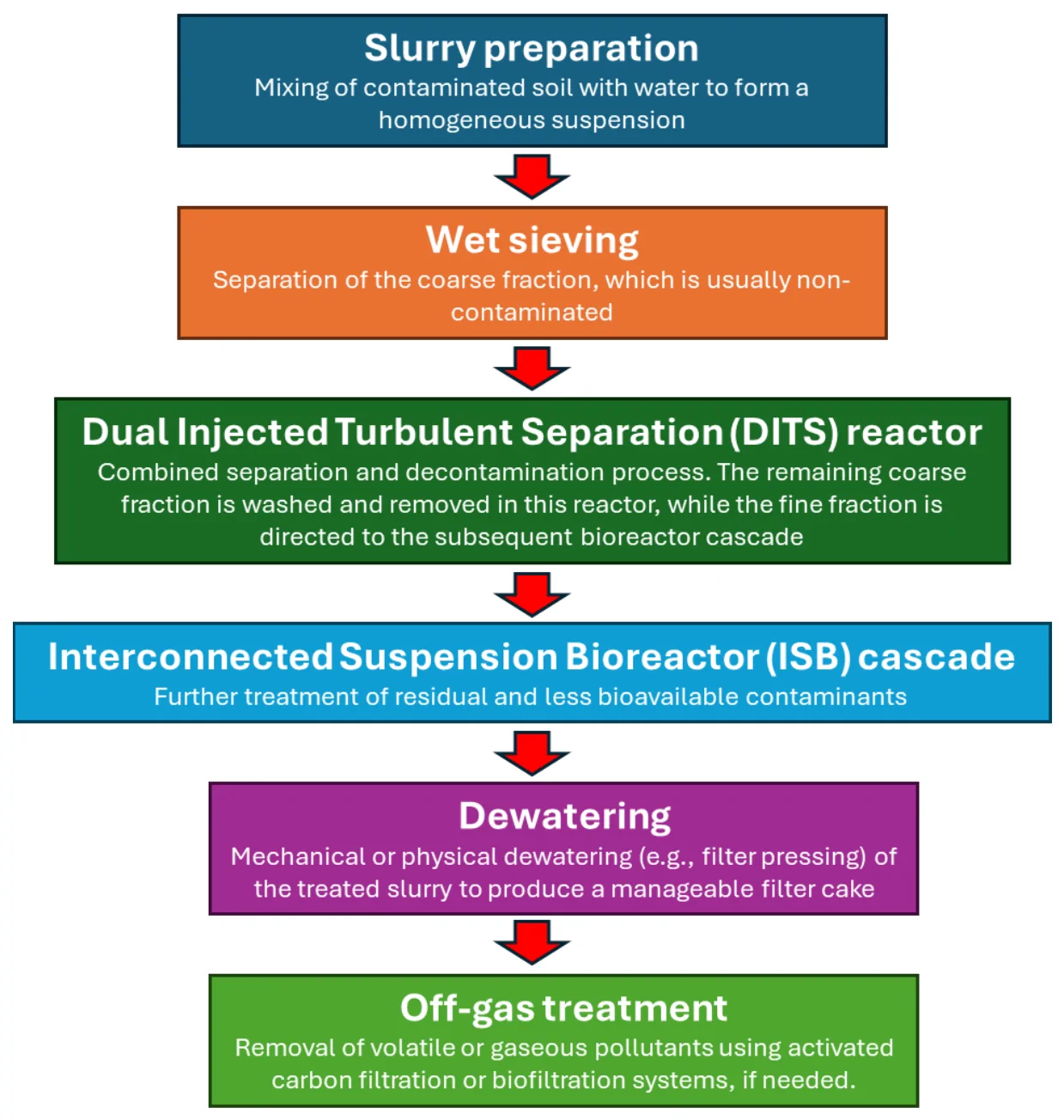
Sequence of operations in a typical slurry-phase treatment plant [46,47].

As mentioned in the previous section, most bioreactors for soil bioremediation are operated in the slurry phase to facilitate the homogeneous mixing of the polluted material with microorganisms and reagents, and to promote exchange processes and reaction kinetics. However, extremely fine-grained, cohesive soils and sludges, such as those generated from pretreatment processes like soil washing, cannot be effectively treated using heap or solid-state bioreactor techniques. Slurry-phase treatments are typically operated in impeller-driven mixing vessels, airlift reactors, or fluidized-bed systems, all of which ensure the adequate and homogeneous distribution of oxygen and nutrients throughout the slurry. The principal operational parameters of slurry-phase bioreactors are described in Table 2, while a scheme of a typical slurry-phase treatment sequence is reported in Figure 2.

Bioreactor design for PTE remediation is essential for scaling up processes from shake flasks to pilot or industrial scale. Design criteria include the culture requirements of the microorganism, operational simplicity, scalability, material demands, and cost-effectiveness [43]. In general, a bioreactor represents both an engineering and bioengineering challenge [44].

According to Morin [45], the design of a bioreactor unit that balances performance with practical considerations is influenced by several key parameters, including maximum head pressure, stirring system size, and reactor configuration. In addition, the design of a bioleach tank circuit at an industrial scale involves compromises based on criteria such as minimizing the number and volume of tanks, retention time, interchangeable tanks, secondary and tertiary stages, and gravity transfer.

Engineers must conceive the physical system to provide optimal conditions for gas, liquid, and solid transport while accommodating the biological processes occurring inside. Modern bioreactor engineering has dramatically improved, enabling faster process development, pretreatment handling, and utilization of renewable raw materials across a wide range of bioprocesses, including large-scale bioreactors and waste treatment plants [44].

Gas–liquid and solid–liquid mass transfer is a key process in slurry bioreactors, as sorption and desorption reactions at the solid/liquid interface play a crucial role in PTE availability. In particular, sorption encompasses surface adsorption and other retention processes, including slow diffusion within soil pores or absorption onto OM, whereas desorption represents PTE release from the solid matrix to the liquid phase [14].

Key parameters influencing sorption/desorption, and therefore PTE leaching, include PTE biogeochemistry, soil pH, texture, OM content, redox conditions, and mineralogical composition, as well as soil/water ratio and mixing speed [14].

A low pH enhances metal solubility and desorption, thereby increasing PTE mobility. Coarse-textured soils with low CEC facilitate percolation and reduce sorption of mobilized PTEs but hinder particle dispersion and reduce contact with chemical lixiviants or microorganisms. Organic matter generally promotes PTE immobilization through complexation, although dissolved OM (DOM) can form mobile metal–organic complexes. Redox fluctuations strongly influence the stability of Fe/Mn oxides and sulfides. Reducing conditions favor PTE release from Fe/Mn oxides, while oxidizing conditions promote sulfide dissolution. Soil mineralogy, especially clay minerals, carbonates, and the above-mentioned Fe/Mn oxides, further regulate sorption and precipitation/dissolution processes. These properties act in combination with microbiological and hydrological drivers to determine the extent of PTE leaching from polluted soils [48].

Mixing devices, both mechanical and pneumatic, maintain solids in suspension, enhance turbulence, and increase mass transfer rates in BSRs. Mixing can be continuous or intermittent, the latter reducing energy costs but providing lower intensity. Effective mixing ensures slurry homogeneity, optimal aeration in aerobic BSRs, and improved adsorption/desorption of pollutants, which collectively enhance bioremediation rates and efficiency. The mixer selection depends on slurry properties and kinetic requirements, and are often determined via pilot studies. Power consumption is a critical factor, as denser slurries require more energy and pose challenges for oxygen transfer in aerobic systems. Therefore, strategies to reduce costs also include intermittent mixing and slurry dilution [36].

BSRs are usually employed for the bioremediation of soils contaminated with recalcitrant organic pollutants under controlled conditions, and also to assess the feasibility and potential of biological remediation strategies for restoring contaminated soils or sites [36]. Although a limited number of applications have been recorded so far, BSRs can be also successfully used to remediate PTE-contaminated soils, favoring either PTE leaching or PTE immobilization, the former being preferred.

White et al. [49] demonstrated that sulfuric acid generated by sulfur-oxidizing microorganisms in slurry bioreactors could effectively leach several metals (Cd, Co, Cr, Cu, Mn, Ni, and Zn) from artificially contaminated soils, although Pb solubilization proceeded slowly and remained incomplete even after 180 days. In a similar study, Gómez and Bosecker [50] observed successful removal of PTEs from contaminated soils using *Thiobacillus ferrooxidans* and *Thiobacillus thiooxidans* under slurry conditions. These authors observed that, depending on the growth medium, soil characteristics, and contaminant type, native isolates were able to solubilize more than half of the PTEs present, including As, Cd, Co, Cr, Cu, Ni, V, Zn, and Be. In particular, complete removal of Cd, Co, Cu, and Ni was achieved with *T. ferrooxidans*, while sulfur-oxidizing strains mobilized over 80% of Cd, Co, Cu, and Zn from rainwater sludge.

Zagury et al. [51] showed that semi-continuous stirred-tank reactors enriched with indigenous iron-oxidizing microorganisms could solubilize substantial proportions of Zn, Cu, and Mn from contaminated soils, reaching 40%, 47%, and 34% extraction, respectively. Nareshkumar et al. [52] demonstrated broad bioleaching capabilities using *Acidithiobacillus thiooxidans* in slurry bioreactors, with Cr, Zn, Cu, Pb, and Cd solubilization ranging from 11% to 99%. Using a different microbial strategy, Soda et al. [53] applied a dissimilatory arsenate-reducing bacterium in a slurry bioreactor, achieving an As removal efficiency of 63% and 41% from artificially contaminated forest and paddy soils, respectively, and 20% and 55% from two naturally contaminated soils. Kumar and Nagendran [54] reported the alteration of PTE-binding forms during bioleaching of metal-contaminated soils using *Acidithiobacillus thiooxidans* in shake flask experiments, and a good removal efficiency of Cr bound to non-reactive soil fractions (organic and residual) was achieved. Yang et al. [55] tested bioleaching using *Burkholderia* sp. Z-90, a biosurfactant-producing strain, obtaining a metal removal efficiency of 44% for Zn, 32.5% for Pb, 52.2% for Mn, 37.7% for Cd, 24.1% for Cu, and 31.6% for As. Furthermore, Mn, Zn, and Cd were more readily mobilized than Cu, Pb, and As, which was largely due to the strain capacity to adhere to contaminated soil particles and form metal–biosurfactant complexes. More recently, Sur et al. [56] showed that applying *Thiobacillus ferrooxidans* to contaminated soils enabled an effective bioleaching within short treatment periods, i.e., after 12 h, with removal efficiencies ranging from 29–76% for Cu, 10–32% for Pb, 39–72% for Cr, and 44–68% for Ni. Details about the type of microorganisms used in BSRs and their reactions are described in the next section.

Therefore, BSRs are potentially applicable to soils and sediments contaminated with a broad range of PTEs. In particular, the most convenient targets for bioleaching are PTEs that occur in relatively labile, exchangeable, carbonate-bound, oxide-associated, or otherwise acid-soluble fractions, because these forms are more readily mobilized by microbial production of acids and other lixiviants. On the contrary, PTEs strongly incorporated into resistant mineral phases are poorly removed and thereby imply additional or alternative treatment strategies [41,57].

Another crucial issue of soil leaching for the remediation of PTEs in BSRs is the formation of a secondary wastewater stream containing the PTEs mobilized from the soil matrix. Unlike organic contaminants, PTEs cannot be degraded; therefore, their elimination from soil via leaching simply represents a transfer of contaminants from the solid phase (soil) to the liquid phase (effluent). Although increasing the liquid-to-solid ratio may improve PTE extraction, these conditions can also increase the volume of wastewater generated and the associated requirements for downstream treatment [58,59]. The obtained leachate may indeed contain high concentrations of dissolved or complexed PTEs, residual lixiviants, and other dissolved soil constituents, often imposing the necessity for its treatment. Hence, the efficiency of soil leaching should not be assessed merely by relying on the reduction of PTE concentrations in the treated soil, but a comprehensive evaluation should also consider the overall mass balance of PTEs, the volume and composition of the generated wastewater, and the feasibility of recovering and recycling the leachate. Efficient treatment or recovery of PTEs from the effluent is vital to prevent secondary pollution and to enhance the environmental and economic sustainability of the remediation process [58,59].

## 4. Microorganisms in PTEs Bioleaching

### 4.1. Physiological and Biochemical Adaptations of Microorganisms to PTE Stress

When dealing with microorganisms for PTE soil remediation, particular attention must be paid to the PTE concentration, as high concentrations of PTEs may significantly affect microbial diversity, community structure, and metabolic activity. Concentrations above which PTEs can become toxic to microorganisms depend on the type of PTE (essential or non-essential), their speciation and bioavailability, and the type of microorganism (susceptible or tolerant). Susceptible species may decline, while tolerant species increase, often accompanied by upregulation of resistance mechanisms [60].

Based on their biological roles in microorganisms, PTEs can be categorized into essential or non-essential elements (Table 3) [61,62].

Microorganisms can avoid PTE toxicity through a variety of passive and active mechanisms. Passive systems reduce intracellular metal exposure without involving the response of the expression of specific genes. Examples include complexation of metal ions with sulfate in acidic environments, chemiosmotic barriers in acidophiles that restrict metal entry, competition of metals with protons for cell surface binding sites, and biofilm formation that sequesters and immobilizes metals [60].

Acidophiles are notable for their high metal resistance, which is partly due to intrinsic tolerance linked to the acidic environments they colonize, rather than a higher abundance of specific resistance genes. At low pH, many metals exist as soluble toxic ions, yet acidophiles efficiently withstand such conditions through both passive and active resistance mechanisms. The latter involve intracellular metal-binding proteins and metal-transforming enzymes, encoded by metal resistance genes. Lateral gene transfer (LGT) facilitates the spread of resistance traits within microbial communities, thus contributing to genome evolution and adaptation to PTEs stress [60].

Another strategy that microorganisms use to survive in the presence of high concentrations of PTEs is by regulating their metal homeostasis. In particular, they maintain metal homeostasis via metal-regulatory proteins, which detect intracellular metal levels and regulate the expression of transporters, metallochaperones, and other proteins involved in metal influx or efflux. Binding of metal ions to these regulatory proteins induces conformational changes that modulate transcription of downstream genes, thus ensuring cellular metal balance [63].

### 4.2. Metabolic Features Underlying PTE Bioleaching

Bioleaching is the process mostly exploited in BSRs to remove PTEs from polluted soils. Bioleaching utilizes the natural metabolic capabilities of microorganisms to solubilize and mobilize metals, making them extractable from contaminated soils. Microorganisms employ both autotrophic (e.g., sulfur- and iron-oxidizing bacteria) and heterotrophic (e.g., biosurfactant- and siderophore-producing bacteria) pathways to promote metal solubilization. In the heterotrophic pathway, organic acids such as oxalic, gluconic, and malonic acids are secreted, lowering pH to 4–6 and enhancing metal solubility through chelation. Since microbial metabolites play central roles in the oxidation, solubilization, and mobilization of PTEs [41]; optimizing bioleaching efficiency requires understanding these mechanisms and tailoring microbial activity towards specific PTEs, or combinations of PTEs, in soil. As bioleaching is usually conducted under non-sterile conditions, native microbial communities can be identified, in laboratory-scale experiments, as novel and specially adapted microbes suitable for bioleaching applications [64].

Bioleaching depends on several physicochemical and biological factors, including the presence of suitable microbial species capable of solubilizing PTEs, the availability of nutrients to stimulate whole or specific microbial communities, and the maintenance of optimal pH, temperature, carbon dioxide, and oxygen supply. Therefore, the effectiveness of bioleaching requires a comprehensive understanding of these factors. For instance, the absence of nutrients compromises microbial activity, making the addition of various organic and inorganic nutrients necessary to enhance bacterial growth in PTE-contaminated soils. Moreover, bacterial solubilization of toxic metal(loid)s is strongly pH-dependent, with low pH required for metals to remain soluble and for Fe/S-oxidizing bacteria and archaea to thrive [41]. Optimizing oxygen and carbon dioxide concentrations further supports microbial growth and enhances PTE extraction yields. Despite increasing research on bioleaching, comprehensive reviews critically evaluating such optimization needs and mechanisms in PTE-contaminated soils are still limited.

Microbial metabolites are generally categorized into primary and secondary ones [65]. Primary metabolites, such as amino acids, carbohydrates, nucleotides, and fatty acids are essential for microbial growth. Secondary metabolites, including antibiotics, pigments, siderophores, and toxins, are produced in the stationary phase and often have ecological, adaptive, or defensive roles [65,66]. These secondary metabolites, unlike primary metabolites, are not required for survival but contribute to environmental adaptation (including PTEs-rich environments) and competitive interactions. In particular, many biosurfactants, which are important in bioremediation management, are products of secondary metabolism [66]. The production of secondary metabolites is tightly dependent on global (pleiotropic) and pathway-specific regulators. Environmental cues, such as nutrient limitation (e.g., nitrogen, phosphate), stressors (pH, temperature, oxidative stress), and signalling molecules like N-acetylglucosamine, trigger these regulatory systems, which control the expression of biosynthetic gene clusters (BGCs) responsible for metabolite production [67]. These BGCs often include genes for self-resistance and efflux systems, such as ATP-binding cassette (ABC) transporters, to prevent intracellular toxicity and feedback inhibition. For instance, in *Cupriavidus metallidurans*, the Rmet_2229-2234 ABC-type transporter is involved in Zn and Cd resistance in extreme environments [68].

### 4.3. Microbial Siderophores for PTE Mobilization

Among secondary metabolites, siderophores (molecules that show a high affinity for iron) have attracted significant interest for their role in PTEs bioremediation. These compounds not only enhance Fe acquisition but can also form complexes with a wide range of other metals, albeit with lower affinity than Fe, thereby improving microbial tolerance to metal-induced stress and promoting environmentally sustainable metal detoxification [69,70]. PTE mobilization by siderophores is not only favored by direct complexation reactions but also by Fe complexation, which promotes Fe-(hydr)oxides solubilization and therefore indirect PTE release, as PTEs are often associated with iron oxides.

Siderophores are divided into various classes based on the bidentate functional groups that make up the chelating moiety. These groups can be a hydroxamate, a catechol, an α-hydroxyacid, a 2-(2-hydroxyphenyl)-oxazoline, or a fluorescent quinoline chromophore. Siderophores typically have three bidentate groups in the chelating portion, which may be all the same, two the same and one different, or all different [71].

Bacterial siderophores are mainly categorized as hydroxamic acids, poly-catechol, substituted carboxyl-based, mixed types, and pseudomimetic catecholates (Table 4).

Pseudobactin and pyoverdine are typical siderophores synthesized by bacteria, whereas fungi predominantly synthesize hydroxamate-type siderophores such as rhodotorulic acid and ferrichromes [69].

Hydroxamate siderophores possess the functional group C(=O)/N–(OH)R, where R represents an amino acid or its derivative, providing two oxygen atoms that coordinate with Fe ions to form a bidentate ligand [70]. Each cluster of hydroxamate molecules can generate a hexadentate ligand, resulting in the formation of an octahedral Fe(III) complex. Fungi and some strains of bacteria mainly secrete hydrophilic tris hydroxamate siderophores characterized by high formation constants of ~10^30^ [74].

In catecholate-type siderophores, Fe(III) is chelated through the hydroxyl moieties of the catechol functional groups. The complexation process yields a hexadentate–octahedral structure, with the vicinal dihydroxyl group from each catechol moiety coordinating the Fe(III) ion. These siderophores are typically derivatives of salicylic acid or 2,3-dihydroxybenzoic acid (2,3-DHBA). An example is spirilobactin, a catecholate siderophore produced by *Azospirillum brasilense* under Fe-limiting conditions [70]. Catecholate siderophores form highly stable complexes with Fe(III), with tris-catecholate enterobactin exhibiting a formation constant of 10^49^, far exceeding that of EDTA (~10^25^). Under strongly basic conditions, fully deprotonated catecholates bind Fe(III) much more strongly than hydroxamates, corresponding to 10^19^-fold stronger complex formation. At physiological pH (7.4), however, the binding affinities of hydroxamate and catecholate siderophores become comparable. This arises from the greater acidity of hydroxamates, which increases the fraction of deprotonated molecules available for Fe(III) coordination, allowing effective metal binding under neutral conditions [74].

**Figure 3:**
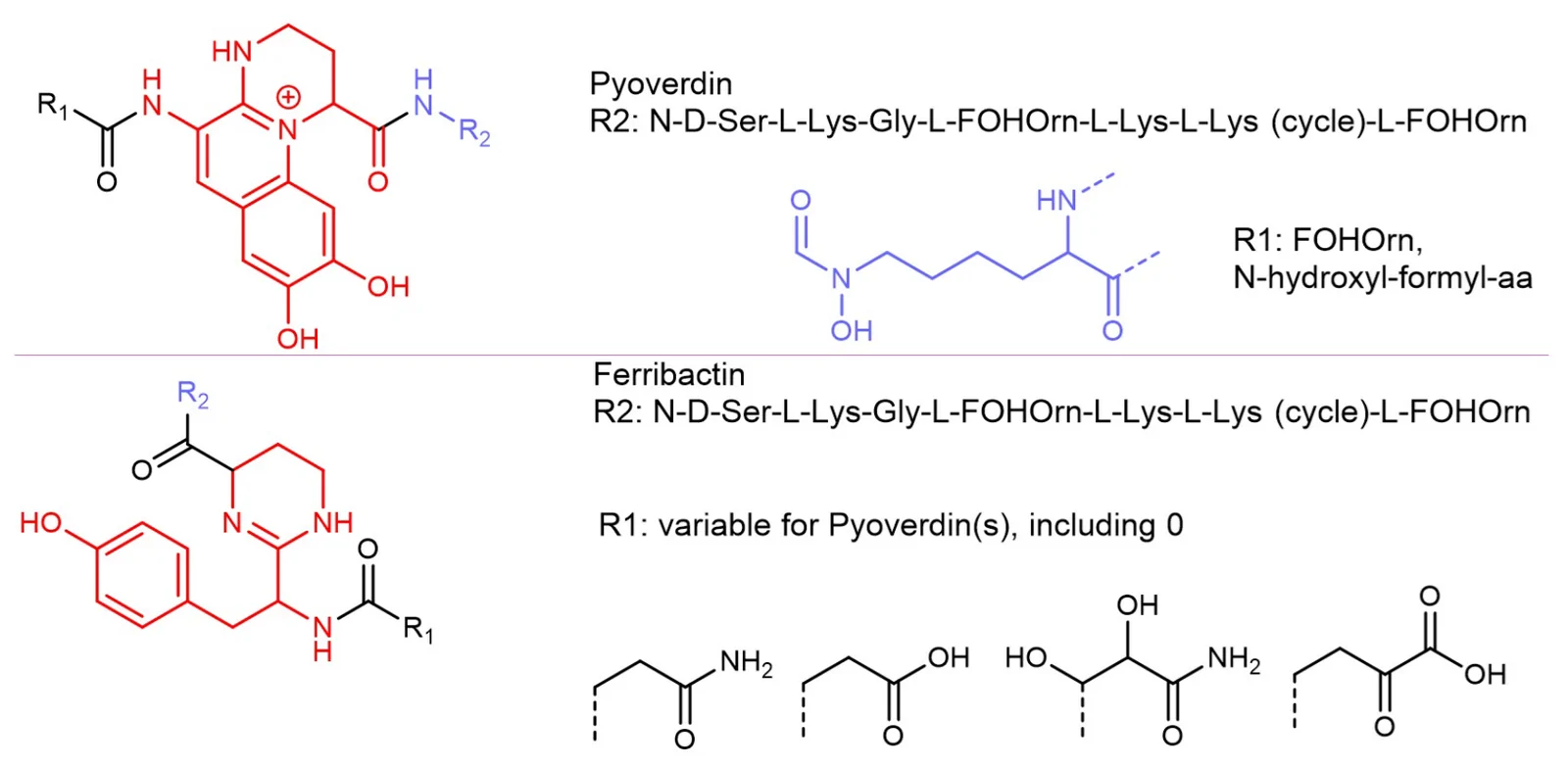
General structures attributed to pyoverdine macromolecules, including pyoverdine(s) and ferribactin produced by Pseudomonas fluorescens. R2 often includes peptidomimetic structural series, with L-Lysine cyclic dimers, while R1 is often a polar-bearing flag moiety. The 2 main categories differ in the macrocycle central ring, referred to as the chromophoric building block.

Hydroxycarboxylate siderophores coordinate Fe through carboxyl and hydroxyl groups and often occur as part of mixed-type siderophores containing multiple Fe-chelating moieties. A well-known group of mixed-type siderophores exhibiting high structural similarity with functional peptides is the pyoverdines, which are synthesized by fluorescent *Pseudomonas* spp. bacteria. Each pyoverdine molecule comprises a quinoline chromophore, a short peptide chain, and a dicarboxylic acid (or its amide) bound to the chromophore. While the peptide sequence remains constant within a bacterial strain, it varies among species and strains. For instance, *Pseudomonas fluorescens* produces several forms of pyoverdine: pyoverdine, pyoverdine 0, and pyoverdine A (ferribactin) (Figure 3) [75]. Pyoverdine is a peptide structurally related to ferribactin, sharing the same overall composition but lacking glutamine and containing a fluorescent chromophore replacing tyrosine [76]. Other Pseudomonas species, including *P. syringae* and *P. aureofaciens*, also synthesize pyoverdine-type siderophores. Moreover, *P. fluorescens* produces additional siderophores such as enantio-pyochelin, quinolobactin, ornicorrugatin, and pseudomonins [70].

Siderophore biosynthesis occurs via two main pathways, i.e., non-ribosomal peptide synthetase (NRPS) and NRPS-independent siderophore (NIS) pathways. The NRPS pathway involves modular enzymatic complexes that activate, assemble, and modify amino acids into highly diverse peptides, while the NIS pathway uses specific enzymes such as IucA/IucC synthetases to condense citrate derivatives with amines or alcohols [77]. Genes encoding siderophore biosynthesis, regulation, and transport are usually organized in BGCs and regulated by Fe-responsive proteins, including ferric uptake regulator (Fur) and diphtheria toxin regulator (DtxR), which ensure homeostasis and efficient siderophore production [77].

Overall, integrating microbial chelating compounds, including siderophores, into bioleaching and bioremediation strategies represents an effective approach to address PTE pollution in soils by combining eco-friendly technology with microbial ecological processes to restore soil health and functionality [41,65,69]. In particular, when used in bioreactor technologies, bioleaching processes promoted by bacteria siderophores can extract PTEs from polluted soils in a controlled and isolated system, thus allowing PTE collection and secondary treatment while recovering a cleaner soil.

### 4.4. Microorganisms Used in PTE Bioleaching

As already mentioned in previous sections, both autotrophic and heterotrophic microorganisms can be exploited for the bioleaching of PTEs from polluted soils. In particular, autotrophic bioleaching utilizes acidophilic, aerobic, chemolithotrophic microorganisms, including both bacteria and archaea, which oxidize inorganic sulfur compounds and ferrous ions to produce sulfuric acid and ferric ions, which are the primary agents driving metal solubilization [57,78]. These microbes fix carbon via the Calvin–Benson cycle and are commonly found in acidic, sulfide-rich environments. Among bacteria, the genus *Acidithiobacillus* is the most extensively studied due to its high tolerance to PTEs and minimal nutrient requirements for metal mobilization [41,79].

*Thiobacillus* spp. are the most effective microorganisms used in bioleaching and thrive under aerobic conditions. The microorganisms belonging to the genus *Thiobacillus* are Gram-negative, non-spore-forming bacteria. Moderately thermophilic bacteria such as *Thiobacillus*-like bacteria (Th-bacteria) use Fe^2+^ to produce energy. They thrive at a temperature limit of 50 °C on pyrite, pentlandite, and chalcopyrite, and yeast extract is essential for their growth [80]. Most thiobacilli are chemolithoautotrophic species; bioleaching occurs at an acidic pH ranging between 1.5–3, where most metals are available in solution. Hence, the acidophilic species *Thiobacillus ferrooxidans* and *T. thiooxidans* are of peculiar significance, whereas other thiobacilli that oxidize sulfur and sulfides proliferate only at higher pH values where metals are often not available in solution [80].

Other microorganisms used in PTE bioleaching belong to the family Sulfolobaceae, within the order Sulfolobales. They are metal sulfide-oxidizing bacteria, either motile or non-motile cocci (single or pairs), that grow at temperatures within the range of 65–90 °C and pH 2. Such cells are characterized by being Gram-negative, highly irregular in shape, and frequently lobed or edged. Their growth can be aerobic, facultatively anaerobic, or anaerobic. Additionally, growth conditions are either autotrophic or heterotrophic. While oxidation of elemental sulfur, thiosulfate, metal sulfides, or molecular H_2_ helps them to obtain energy under autotrophic conditions, heterotrophic growth is maintained by aerobic respiration, anaerobic sulfur respiration, or fermentation of organic substrates [81,82,83,84].

*Leptospirillum ferrooxidans*, an acidophilic obligatory chemolithotroph, depends on Fe^2+^ to obtain energy. As compared to *Acidithiobacillus ferrooxidans*, this bacterium possesses higher endurance at a lower pH (about 1.2), as well as at higher concentrations of PTEs such as U, Mo, and Ag. However, it is highly sensitive to Cu and does not possess the ability to oxidize sulfur compounds. *A. ferrooxidans* is widely spread in the environment and proliferates at higher rates than *L. ferrooxidans*, but Zn^2+^ addition can stimulate *L. ferrooxidans* growth [85,86]. For *Leptospirillum* spp. growth, a temperature range of 20–45 °C is almost tolerable [86,87].

Heterotrophic microorganisms rely on the indirect production of organic acids, biosurfactants, and other metabolites to solubilize metal ions. Common organic acids include oxalic, gluconic, lactic, acetic, succinic, pyruvic, malonic, isocitric, and formic acids [41], which facilitate metal solubilization by maintaining low pH, promoting electron transfer, and forming soluble metal complexes. Key heterotrophs for bioleaching include *Aspergillus niger* and *Penicillium simplicissimum*, which are known for excreting significant quantities of organic acids and siderophores. Bioleaching using fungi (*Penicillium* and *Aspergillus* spp.) and sulfur- and iron-oxidizing bacteria has shown promising results for the remediation of Cd- and As-contaminated paddy fields [41]. Tran et al. [88] reported that the use of *Shewanella putrefaciens* with indigenous bacteria removed 57.5% of As from contaminated agricultural soil, compared to 16.4% and 30.1% removal by indigenous bacteria and *S. putrefaciens* alone, respectively.

The genera Bacillus and Pseudomonas are highly effective for bacterial bioleaching, while Aspergillus and Penicillium dominate among fungi. Deng et al. [89] demonstrated that *Penicillium chrysogenum* strain F1 achieved removal efficiencies of 74%, 59%, 24%, 55%, 57%, and 25% for Cd, Cu, Pb, Zn, Mn, and Cr, respectively, outperforming chemical leaching. *Aspergillus niger*-derived organic acids have also exhibited enhanced removal of toxic metal(loid)s from industrially contaminated soils [41]. Therefore, bioleaching using soil heterotrophic microorganisms provides an eco-friendly approach for extracting PTEs from contaminated soils.

Table 5 summarizes the various microorganisms involved in bioleaching processes, which includes bacterial, archaeal, and fungal species, also highlighting their type of metabolism, optimal temperature and pH conditions, target PTEs, and their ability to oxidize Fe or S.

Genetically modified microorganisms (GMMs) represent a promising emerging approach for improving bioslurry-based PTE bioleaching. Bioleaching relies on sustained microbial production of leaching agents, such as protons, ferric iron, sulfuric acid, organic acids, and metal-complexing metabolites, which induce the dissolution and mobilization of PTEs from the solid phase into the aqueous phase [41,57]. However, bioleaching efficiency is often limited by the sensitivity of microorganisms to high concentrations of dissolved metal ions, prolonged exposure to acidic conditions, and other physicochemical stresses that can inhibit microbial growth and metabolism. Genetic engineering could potentially address these limitations by enhancing metal tolerance and stress resistance and by improving metabolic pathways associated with Fe and S oxidation or the production of biogenic leaching agents. Chen et al. [102] highlighted the potential of genetically engineered *Acidithiobacillus* spp. to enhance biomining performance, including ameliorated tolerance to harsh conditions and enhanced metal extraction, while recent reviews have demonstrated gene editing and synthetic biotechnology as promising approaches for establishing bioleaching microorganisms with greater resistance to metal-ion stress and better leaching efficiency [102,103]. Engineered yeast strains have been shown to sequester metals, including Cr, As, and Cd, at concentrations 10–100 times higher than typical hyperaccumulator thresholds [104].

Hence, in a bioslurry reactor, GMMs could potentially attain higher metabolic activity under conditions that limit the activity of conventional strains, leading to more sustained production of lixiviants and enhanced PTE mobilization. Genetic engineering may also enable the development of strains addressed to specific contaminated matrices or target PTEs, potentially enhancing the selectivity and efficiency of bioleaching. However, the application of GMMs for PTE bioleaching remains largely at the laboratory and proof-of-concept stages, and significant challenges include the stability of engineered traits, maintenance of performance under complex slurry conditions, and biosafety and regulatory requirements, as well as the establishment of efficient containment strategies. BSRs may provide an advantage in this respect because its ex situ and controlled operation could facilitate containment and monitoring compared with direct environmental application. Consequently, genetically engineered bioleaching microorganisms should currently be viewed as an emerging strategy with considerable potential for improving PTE mobilization and extraction but necessitating further validation under realistic bioslurry conditions.

### 4.5. Mechanisms of PTE Mobilization

In bioleaching, microorganisms remove metal ions from the contaminated soil via two main mechanisms, namely indirect and direct bioleaching. In the indirect mechanism, bacteria do not physically contact the solid substrate but promote PTE solubilization through the production of metabolites. In direct bioleaching, bacteria interact with the solid surface, facilitating metal solubilization through electrochemical reactions [42].

In indirect bioleaching, chemoautotrophic Fe-oxidizing microorganisms like *Acidithiobacillus ferrooxidans* or *Leptospirillum ferrooxidans* generate ferric ions by oxidation of ferrous iron [105,106]. Ferric iron then acts as a chemical oxidizing agent that disrupts the bonds, and therefore the structure, of PTE-containing minerals, mainly sulfides. Another mode of action involves a confined reaction zone between the microbial cell wall and the mineral surface, where ferric ions accumulate within biofilms to target the sulfide mineral locally [106]. Under natural conditions, ferrous-to-ferric iron oxidation is extremely slow; however, microbial activity accelerates this reaction by 10^5^–10^6^ times at pH 2–3 [107].

Acidophilic bacteria oxidize sulfide minerals such as pyrite, producing energy for microbial growth. Oxidation of sulfides then generates sulfuric acid, which further acidifies the environment and enhances metal dissolution. Thus, metals such as Cu, Zn, and Ni are released from minerals as soluble sulfate complexes in the acidic solution. Indirect bioleaching disrupts passivation layers on mineral surfaces, exposing fresh material for further oxidation and metal release. Microbes then continue to grow, sustaining ferric ion production and sulfuric acid generation over time.

Regarding the direct bioleaching mechanism, [108] described the following processes occurring during PTEs mobilization: (1) bacteria attachment to PTE-mineral surfaces; (2) oxidation of Fe and S, producing Fe^3+^ and SO_4_^2−^; (3) acid generation, reaching about pH 2 around mineral particles which further facilitate PTEs dissolution; (4) PTE complexation with bacterial ligands, facilitating their transport away from the mineral matrix; (5) PTE uptake by bacteria possessing ion exchange and transport mechanisms allowing them to uptake metal ions into the cells; (6) bacterial SO_4_^2−^ reduction to generate energy, producing S^2−^ and additional H^+^ that maintain the acidic conditions necessary for continued metal solubilization.

In these processes, both oxidation and reduction reactions sustain bacterial growth, maintaining a stable microbial population that drives the bioleaching processes. In addition, under certain conditions, metal ions within bacterial cells may precipitate as metal sulfides, which can be advantageous for recovering metals from solution.

Over the past few decades, a particular interest around bioleaching mechanisms has grown, especially by the metal recovery industry, as the microbial extraction of PTEs from sulfidic ores has become an established biotechnology [80,105,109,110,111,112,113].

Since metal sulfides can be divided into two distinct groups, two different oxidation mechanisms have been proposed: (i) the thiosulfate mechanism, which applies to acid-insoluble sulfides such as pyrite, and (ii) the polysulfide mechanism, which is relevant for acid-soluble sulfides like sphalerite (ZnS) or chalcopyrite (CuFeS_2_) [84].

The thiosulfate pathway for acid-insoluble metal sulfides such as pyrite, molybdenite, and tungstenite (FeS_2_, MoS_2_, and WS_2_, respectively) requires an oxidation process for their dissolution. The chemical bonds between the metal and sulfur atoms are not broken until six successive one-electron oxidation steps occur, which result in the formation of thiosulfate (S_2_O_3_^2−^; [113,114,115,116]) (Equation (1)). Under bioleaching conditions, Fe(III) ions serve as the primary oxidant, accepting electrons mainly from the sulfide and being reduced to Fe(II) ions. Because the first free sulfur compound formed is thiosulfate, this process is referred to as the “thiosulfate pathway” [113,116].

FeS_2_ + 6 Fe^3+^ + 3 H_2_O → S_2_O_3_^2−^ + 7 Fe^2+^+ 6 H^+^ (1)

Thiosulfate is then predominantly oxidized via tetrathionate, disulfane monosulfonic acid, and other polythionates to sulfate. However, significant amounts of elemental sulfur (10–20%) may form if bacterial S-compound oxidation is absent, which alternatively occurs through purely chemical oxidation by Fe(III) ions (Equation (2)) or by *Leptospirillum ferrooxidans* [113,117,118].

S_2_O_3_^2−^ + 8 Fe^3+^ + 5 H_2_O → 2 SO_4_^2−^ + 8 Fe^2+^ + 10 H^+^ (2)

Since electron extraction relies on Fe(III) ions, and Fe(II) ions are not significantly oxidized abiotically at around pH 2 [113,119], only Fe(II)-oxidizing bacteria, such as *Acidithiobacillus ferrooxidans* and *Leptospirillum ferrooxidans*, can efficiently leach acid-insoluble metal sulfides under acidic conditions. These microorganisms regenerate Fe(III) ions consumed during the initial oxidation, making the leaching rate largely dependent on bacterial Fe(II) oxidation activity [113,120]. In the absence of bacteria and under acidic conditions without significant Fe(III) ions, pyrite leaching rates are drastically reduced [113,117,118]. Nevertheless, even under such conditions, the thiosulfate mechanism appears to operate, although subsequent thiosulfate reactions may differ from those described above [113,121]. The occurrence of the thiosulfate pathway is confirmed in bioleaching experiments using *A. ferrooxidans* [122,123].

Acid-soluble metal sulfides, such as sphalerite, galena, arsenopyrite, chalcopyrite, and hauerite (ZnS, PbS, FeAsS, CuFeS_2_, and MnS_2_, respectively), are dissolved through a combined action of proton attack and electron extraction by Fe(III) ions, involving removal of valence-band electrons from metal–S bonds. In contrast to acid-insoluble sulfides, these bonds can be disrupted not only by Fe(III) ions but also directly by protons. In Fe-free systems, proton attack could theoretically lead to the release of hydrogen sulfide (H_2_S), as may occur for pure ZnS. However, Fe(III) ions are almost invariably present as impurities and promote simultaneous one-electron oxidation of the sulfur moiety during proton attack. As a result, the first free sulfur species formed is most likely a sulfide cation (HS^+^), which readily dimerizes to disulfide (H_2_S_2_) and is subsequently oxidized via higher polysulfides and polysulfide radicals to elemental S [113,124], according to Equations (3) and (4). This reaction sequence is known as the “polysulfide pathway” [125].

MS + Fe^3+^ + H^+^ → M^2+^ + 0.5 H_2_S_n_ + Fe^2+^ (n ≥ 2) (3)

0.5 H_2_S_n_ + Fe^3+^ → 0.125 S_8_ + Fe^2+^ + H^+^ (4)

In the absence of S-oxidizing microorganisms, more than 90% of the sulfide-S is converted to elemental S during polysulfide oxidation, while minor amounts of thiosulfate, polythionates, and sulfate are also formed [113,124,125].

Acid-soluble metal sulfides may also be leached by S-oxidizing bacteria. Under Fe(III)-limited conditions, these microorganisms oxidize free sulfide produced by proton attack through elemental S to sulfuric acid, thereby regenerating protons consumed during mineral dissolution [113], according to Equation (5).

0.125 S_8_ + 1.5 O_2_ + H_2_O → SO_4_^2−^ + 2 H^+^ (5)

The polysulfide mechanism thus plays a central role in the dissolution of acid-soluble metal sulfides under acidic conditions, where sulfide is oxidized to elemental S via polysulfide intermediates [123,125]. The reaction sequence leading to elemental S formation through polysulfides has been widely documented [123,126,127]. Consistent with these findings, S- and O-containing oxidation products have been identified during bioleaching of sphalerite and chalcopyrite by *Acidithiobacillus ferrooxidans* [128,129], as cited in Pattanaik et al. [123].

Operational parameters modulate the relative contribution of the polysulfide and thiosulfate pathways by controlling proton activity, Fe^3+^/Fe^2+^ cycling, microbial oxidation rates, and the accessibility of sulfide surfaces. First of all, pH governs proton-promoted cleavage of acid-soluble sulfides and the stability of Fe^3+^, thereby influencing progression through polysulfide intermediates toward elemental S and sulfate. For acid-insoluble sulfides, pH primarily affects Fe^3+^ persistence and the subsequent oxidation of thiosulfate to higher-valence S-species [130]. Redox potential (Eh) determines the Fe^3+^/Fe^2+^ ratio and thus the oxidative capacity of the system. High Eh sustains Fe^3+^-driven attack on sulfides and oxidation of polysulfide-derived S. In the thiosulfate pathway, an oxidizing Eh favors Fe^3+^-mediated formation and stepwise oxidation of thiosulfate [64]. Temperature modulates microbial kinetics within the physiological optimum, with elevated temperatures accelerating Fe^2+^ oxidation, S oxidation, and overall reaction rates in both pathways; supra-optimal temperatures suppress microbial regeneration of Fe^3+^ and S oxidation [86,131]. Additionally, oxygen availability controls aerobic Fe- and S-oxidizing microbial activity. Adequate O_2_ ensures continuous Fe^2+^ → Fe^3+^ regeneration and oxidation of reduced S-species, while oxygen limitation reduces Fe^3+^ availability and slows thiosulfate formation and turnover [130]. The Fe^3+^/Fe^2+^ ratio is another key determinant of oxidative dissolution: high Fe^3+^ concentrations enhance attack on sulfides and sustain polysulfide oxidation. For acid-insoluble minerals (e.g., pyrite), Fe^3+^ is the primary oxidant generating thiosulfate, which is subsequently oxidized by microbial activity [64]. Particle size influences reactive surface area and mass-transfer efficiency. Finer particles enhance Fe^3+^ and microbial contact, increasing formation and oxidation of polysulfide and thiosulfate intermediates [131]. Slurry density affects oxygen transfer, dissolved-metal accumulation, and microbial inhibition, with excessive solids reducing Fe^3+^ regeneration and limiting oxidation rates in both pathways [86].

In practice, slurry stirring improves mass transfer of O_2_, Fe^3+^, and soluble S-species, thus sustaining continuous oxidation of polysulfides and thiosulfate, while longer residence allows complete oxidation of polysulfide-derived S and thiosulfate intermediates to sulfate, enhancing overall metal dissolution.

Heterotrophic bacteria and fungi, which rely on organic nutrients for growth and energy, can facilitate PTE mobilization either through enzymatic reduction of highly oxidized metal compounds or by secreting organic acids such as lactic, oxalic, citric, and gluconic acids and compounds with multiple hydrophilic reactive groups, including phenol derivatives [108]. Organic acids are employed by heterotrophic microorganisms to mobilize PTEs via two main mechanisms, i.e., acidolysis and complexolysis. In particular, acidolysis involves protonation of oxygen atoms on insoluble PTE compounds, thus increasing PTE solubility. The degree of protonation depends on the acid strength, making acid biosynthesis crucial for efficient PTE solubilization. Acidolysis is rapid and common for fungi and heterotrophic organisms like *A. ferrooxidans* and *L. ferrooxidans*. Complexolysis, or ligand-induced PTE solubilization, occurs when PTEs interact with microbial chelators at the surface of PTE-bearing phases, thus releasing PTEs from the mineral lattice or from OM. Among these chelators, oxalic acid forms complexes with Al, Fe, and Mg, while citric acid forms complexes with Ca and Mg. Therefore, these molecules facilitate the dissolution of soil minerals such as (hydr)oxides and carbonates, PTE leaching efficiency being then influenced by the complex stability and ligand strength. Microorganisms involved in complexolysis include *Chromobacterium violaceum*, *Pseudomonas aeruginosa*, *Pseudomonas fluorescens*, and *Bacillus megaterium* [41].

Among the metabolites produced by heterotrophic microorganisms, biosurfactants play also an important role in PTE mobilization and are increasingly applied in environmental remediation due to their biodegradability and eco-friendly nature. Biosurfactants contain hydrophilic polar groups (oligosaccharides, monosaccharides, proteins, or peptides) and hydrophobic moieties (saturated or unsaturated fatty alcohols or hydroxylated fatty acids), which confer high surface activity and strong tolerance to environmental stress, making these molecules highly effective for mobilizing metal ions from soil particles.

Two main mechanisms have been identified for biosurfactant-mediated PTE desorption from contaminated soils. First, biosurfactants can form complexes with free ionic PTE species. According to Le Chatelier’s principle, complexation reduces the solution-phase activity of PTE ions, increasing their desorption from soil. Second, biosurfactants can accumulate at the solid–solution interface, lowering the interfacial tension and promoting PTE complexation. The strong interactions between anionic biosurfactants and metal ions form stable complexes, which are often stronger than PTE–soil interactions. These neutral metal–biosurfactant complexes can further incorporate PTEs into micelles, facilitating their desorption into the soil liquid phase [132]. Table 6 lists some metabolites, substances, and microorganisms used for PTE bioleaching, and their mechanism of action.

## 5. Immobilized Bacteria

Bacterial immobilization has been widely studied. In many applications, free-floating bacterial cells are typically utilized in bioreactors for bioleaching. Due to their small size (approximately 1–10 μm) and density, the cells are easily dispersed within the medium, but their further separation and recovering from the medium is challenging. To recycle cells for achieving high cell densities or for subsequent processing, centrifugation and other separation techniques are frequently employed. However, these methods often require substantial capital investment and high energy input, and feature the risk of contamination if the recycled cells are reintroduced into the bioreactor. In continuous systems, cell growth is counterbalanced by effluent washout, thus yielding low cell densities that can compromise their efficiency [133].

Whole-cell immobilization represents a solution to these limitations, as it allows the physical confinement or localization of intact cells to a defined region of space while maintaining the desired catalytic activity. Immobilization enables cells to be retained within bioreactors, facilitating high cell densities, and allows their straightforward separation when needed. This concept originates from enzyme immobilization, as both enzymes and whole cells act as biological catalysts from a chemical engineering perspective, also adding important advantages such as entrapment, adsorption, and encapsulation. However, immobilization presents additional challenges, particularly for living cells, which are more complex than enzymes. On the other side, whole cells offer unique advantages, which are unavailable for enzyme systems, such as yeast self-flocculation, aggregation of plant cells, and bacterial biofilm formation. Compared to free cells, immobilized cells, whether attached to surfaces, aggregated, or within biofilms, often exhibit an increased tolerance to environmental stresses. Moreover, advances in genetic engineering allow the introduction of additional desirable traits, such as self-flocculation and transfer between species, which further enhance the potential of immobilized cell technology for BSR applications.

Cell immobilization provides several benefits, primarily through the establishment of a favorable microenvironment, including enhanced cell–cell interactions and nutrient or pH gradients, which can enhance cellular activities. Key advantages include (i) maintenance of high cell densities in bioreactors; (ii) reduced cost and ease of cell separation from culture media; (iii) facilitation of continuous culture processes at high dilution rates; (iv) increased resistance to shear stress, particularly for shear-sensitive cells; (v) shortened lag phase in cell growth; (vi) improved volumetric productivity; (vii) enhanced substrate utilization; (viii) lower risk of microbial contamination; (ix) better tolerance to substrate or end-product inhibition; and (x) improved genetic stability.

Immobilizing a pure culture or laboratory-formed microbial consortium is generally more efficient than using a naturally growing microbial community from complex contaminated environments, which may contain diverse and sometimes antagonistic species.

In particular, studies suggest that immobilizing naturally occurring microbial consortia or sludge can be less efficient than laboratory-formulated consortia, due to the lack of co-metabolic or synergistic degradation pathways. Borrowing an example from biogas production, anaerobic sludge immobilized in macroporous polyvinyl alcohol (PVA) cryogels enhanced methanogenic activity (~55%) but reduced acidogenic activity (~16%) [134].

Immobilized enzymes are also largely employed in bioreactor technologies. While enzyme immobilization is a concept that has existed for over a century, its practical applications have flourished over the last fifty years. Immobilized enzymes have been successfully applied in the synthesis of complex drug intermediates, chemical transformations under mild conditions without toxic by-products, remediation of polluted environments, disease diagnostics, and correction of metabolic deficiencies [135]. Immobilized enzymes allow convenient handling, simplified product separation, reusability, stability under extreme conditions, and adaptability to all reactor types, including continuous and fixed-bed systems. Additionally, immobilized enzymes are particularly suitable for multi-enzyme or chemoenzymatic cascade processes.

Both bacterial and enzymatic immobilization need suitable support materials for successful practical applications. Attachment, entrapment, and encapsulation are widely used techniques for immobilizing whole cells on support materials. These methods are applicable to nearly all viable and nonviable cells of interest, including microorganisms and plant, mammalian, and insect cells. Although many principles of enzyme immobilization apply to cells, the relative importance of each method differs, due to the size and biochemical complexity of cells.

According to Ge et al. [133], the selection of support materials is crucial for effective immobilization. Ideal support materials should (i) be available in large quantities at low cost; (ii) exhibit mechanical, chemical, and thermal stability during operation and storage; (iii) provide large surface areas accessible to cells and substrates; (iv) contain functional groups to facilitate cell binding; (v) maintain cell activity without causing lysis; (vi) be easy to handle, recycle, or dispose of safely; and (vii) feature low affinity for contaminants.

Additional properties, such as porosity, swelling capacity, compressibility, biodegradability, and microbial growth potential, may be optimized for specific applications.

Various support materials have been developed, including polymeric matrices like alginate, agar, gelatine, K-carrageenan, chitosan, pectin, polyacrylamide, epoxy resin, and silica sol, as well as preformed supports like wood chips, stainless steel, cotton cloth, porous glass, diethylaminoethyl (DEAE) cellulose, porous ceramics, and diatomaceous earth. Table 7 shows the criteria that should be adopted while choosing the appropriate bulk material for cell and enzyme immobilization.

### 5.1. Support Materials for Bacteria Immobilization

Carrier materials used for immobilizing cells are generally classified into inorganic materials, natural organic polymers, and synthetic organic polymers. The support matrix must provide adequate structural support and allow efficient mass transfer to achieve an effective bioremediation. Commonly used matrices include ceramics, activated carbon (AC), lignocellulosic biomasses, and natural or synthetic polymers for immobilization or entrapment of bacterial cells. The physical properties of these carriers are critical, as they determine matrix durability. Immobilization on these matrices can also stimulate cellular adaptations that promote attachment and growth within the support. Additionally, the bacterial cell membrane permeability facilitates nutrient uptake, which must remain unaffected after immobilization [134].

Organic carriers are preferred for whole-cell immobilization due to their biocompatibility. Both natural and synthetic polymers have been explored as matrices for bacterial immobilization in bioremediation. Natural organic carriers are readily available, inexpensive, biodegradable, and non-toxic. Their abundant surface functional groups further enhance adsorption capacity. Examples include carrageenan, pectate, agar, agarose, chitosan, charcoal, cellulose, gelatine, collagen, bacterial cellulose, and alginate. Natural polymers are usually gelled through cooling or ion-mediated gelation. Bacteria immobilized in matrices such as carrageenan and alginate maintain growth and viability. Alginate gels, for instance, are permeable and transparent and do not cause significant physicochemical changes to entrapped cells. Chitosan, with its reactive amino groups, enhances bacterial attachment to the support.

Despite these advantages, natural extracted carriers have limitations, including particle disruption under high bacterial density, excessive gas production, low mechanical and chemical stability, poor reproducibility, high contamination risk, rapid hydration, and loss of adhesiveness during relatively long-term storage (~20–25 days).

To overcome these drawbacks, synthetic polymeric gels, synthesized by chemical or photochemical polymerization of monomeric units, are used. Although less biodegradable and possessing lower diffusion than natural matrices, synthetic polymers provide superior mechanical stability. Widely used synthetic gels include polyvinyl alcohol (PVA), polyacrylamide (PAM), polycarbamoyl sulfonate (PCS), and polyethylene glycol (PEG). Common synthetic plastics include polyethylene (PE), polyurethane (PU), polypropylene (PP), polyacrylonitrile (PAN), and polyvinyl chloride (PVC). Additionally, composite matrices combining natural and synthetic polymers provide an optimal balance of mechanical strength and diffusion efficiency.

Hydrogels are particularly attractive for immobilization due to their water retention, swelling ability, and mechanical strength. Cell entrapment in hydrogels is a preferred immobilization method because it maintains high cell viability and imposes minimal stress to the cells [136]. Water within hydrogels generates osmotic pressure that promotes the interaction among pollutants, bacteria, and carrier material, thus making hydrogel-based immobilization highly suitable for bioremediation. Immobilized cells in natural or synthetic polymers have been used to remove hydrocarbons, organic and inorganic dyes, aromatic compounds (e.g., pyridine, toluene), PTEs, and excess nutrients like nitrogen and phosphorus from wastewater [134].

Among hydrogels, alginate, composed of guluronic (G) and mannuronic (M) acid blocks, is produced by the bacteria *Azotobacter* spp. and *Pseudomonas* spp., and seaweed. Calcium alginate, a widely used alginate, forms gels when calcium ions crosslink G blocks, thus forming a sol–gel matrix that protects entrapped cells. Pore sizes of typical 5–200 μm are produced by crosslinking rate and calcium diffusion. Despite excellent biocompatibility and water retention, alginate exhibits poor cell adhesion and is often modified with peptides, inorganic materials, or other polymers to improve its performance [136].

Synthetic polymers allow tailored control over porosity, hydrophilicity, and mechanical properties. Polyacrylamide (PAM) gels have been extensively used due to ease of fabrication, optical transparency, and mechanical tunability via bis-acrylamide crosslinking. Optimization of polymerization time and temperature is essential to maintain cell viability. PVA hydrogels are non-toxic and cost-effective, and can be gelled through repeated freeze–thaw cycles. Crosslinking with boric acid or incorporation of calcium alginate improves mechanical strength while retaining cellular activity. Advanced PVA hydrogels, crosslinked with 4-carboxyphenylboronic acid, offer tunable mechanical properties and show potential in tissue engineering. Polyurethane hydrogels, formed via polyaddition of isocyanates and polyols, allow cell entrapment through urea linkages or polycondensation. Methacrylate-based hydrogels, photo-crosslinked by UV light, are also widely employed with pre-polymers such as polyethylene glycol dimethacrylate and gelatine methacrylate, enabling rapid gelation [136].

### 5.2. Methods for Bacterial Immobilization

Various types of immobilized cell systems exist and can be chosen in BSR technology, based on the intended application and the characteristics of the immobilization matrix. Some of the commonly used techniques are described below.

#### 5.2.1. Entrapment

Entrapment is a versatile method used to confine microbial cells within a porous polymeric matrix, thus enabling substrate and product diffusion while shielding the cells from external stress. Natural and synthetic polymers such as alginate, agarose, and polyacrylamide are frequently used.

In bioremediation, entrapment has been applied to nitrifying sludge cells to mitigate silver nanoparticle and ion toxicity, which are common contaminants in wastewater. Entrapped fungi and yeast have also been used to remove inorganic contaminants. For example, *Aspergillus sydowii* immobilized in magnetic chitosan microspheres absorbs Cu(II), and Saccharomyces cerevisiae entrapped in a graphene oxide/PVA/calcium alginate composite successfully sequesters U through ion exchange, complexation, and reduction [137].

#### 5.2.2. Adhesion and Adsorption

Immobilization through adhesion or adsorption relies on electrostatic forces between the cell membrane and solid supports. Cells can form biofilms on porous or nonporous surfaces, thus acquiring protection and maintaining viability. This method allows direct contact to nutrients and efficient substrate conversion, though weak stabilization forces can result in cell detachment.

Materials such as bacterial cellulose (BC) have been used to immobilize diverse microorganisms, thus demonstrating the importance of cell size, shape, and contact area for successful adsorption [138]. Lapponi et al. [137] reported that *Pleurotus ostreatus* adhered to bentonite achieves >95% PTE removal.

Optimizing adhesion involves controlling support characteristics (e.g., particle size, porosity, reactive groups), cell properties (e.g., surface charge, hydrophobicity, size, age), and environmental factors (e.g., pH, temperature, flow rate). Chemical modifications or composite supports can enhance binding and prevent cell detachment.

#### 5.2.3. Cross-Linking

Cross-linking stabilizes cells by forming covalent bonds between activated supports and cells or macromolecules by means of multifunctional agents such as glutaraldehyde, hexamethylene-diisocyanate, or bis-diazo-benzidine. For instance, *Bacillus pseudomycoides* immobilized in PVA hydrogel cross-linked with glutaraldehyde maintained high viability for nearly two months and efficiently treated municipal wastewater. Cross-linking agents have also been employed to immobilize nitrifying bacteria and other strains for diverse biotechnological applications [134].

Radiation-based cross-linking, including gamma or UV irradiation, offers an alternative that avoids cytotoxic agents while enabling polymerization at room temperature, sterilization, and simultaneous immobilization. Successful applications include immobilization of multiple bacterial strains for Sr remediation and PAH degradation [134].

#### 5.2.4. Covalent Binding

Covalent binding provides strong, stable attachment of cells to surfaces, minimizing detachment. Glutaraldehyde is widely used to anchor cells, induce aggregation, or determine artificial flocculation [137].

**Figure 4:**
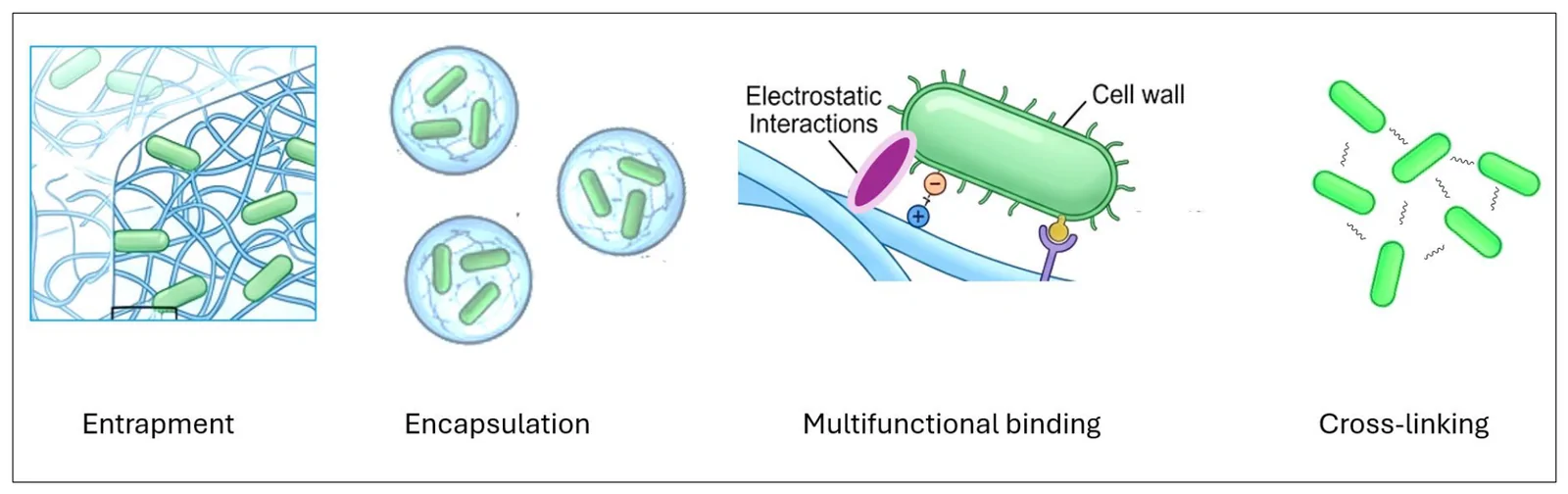
Different methodologies used for whole-cell immobilization (adapted from [137]).

Figure 4 summarizes the different methodologies used for whole-cell immobilization.

The type of immobilization procedure can involve the aforementioned reversible or irreversible interactions, and the choice of interaction depends on the intended application of the immobilized cell system and the specific reaction involved. Additionally, the advantages and limitations of each technology should be carefully considered to achieve the desired outcome [137].

The various types of cell immobilization and their advantages and disadvantages are reported in Table 8.

Despite the potential advantages of microbial immobilization, several challenges persist, including operational complexity, variable results in the presence of multiple contaminants, design considerations for large-scale operations, mass transfer limitations, accumulation of toxic substances or metabolic products near immobilized cells due to slow diffusion, and formation of thick biofilms that can obstruct bead pores and reduce substrate transport to the cells [134]. Although increasing biofilm thickness (L_f_) enhances biomass retention and may therefore increase the potential substrate-conversion capacity, it simultaneously increases the diffusion distance through the biofilm, thus reducing substrate accessibility to microorganisms located in deeper layers. This trade-off is particularly crucial because the heterogeneous structure of biofilms can induce substantial spatial variations in substrate concentration and mass-transfer rates [139]. For a one-dimensional biofilm under steady-state diffusion and first-order substrate utilization, the relative importance of reaction and diffusion can be characterized by the Thiele modulus (φ) (Equation (6)):

(6) $$\varphi =L_{f}\sqrt{\frac{k}{D_{\mathrm{eff}}}}$$

where k is the first-order reaction constant and D_eff_ is the effective substrate diffusivity within the biofilm [140]. The corresponding effectiveness factor for an idealized planar biofilm is given by Equation (7):

(7) $$ŋ=\frac{\tanh(\varphi )}{\varphi }$$

where ŋ represents the ratio of the actual reaction rate under internal diffusion limitation to the reaction rate that would occur if the entire biofilm were exposed to the bulk-substrate concentration [140]. Thus, as L_f_ increases, φ increases and ŋ decreases when internal diffusion becomes increasingly limiting. The observed conversion rate (R_obs_) can consequently be expressed by Equation (8):

**R_obs_** = **ŋ R_intrinsic_** (8)

which implies that an increase in biomass does not eventually result in a proportional increase in reactor performance because an increasing fraction of the biofilm may become substrate limited. In bioslurry reactors, this biofilm-associated resistance is superimposed on contaminant desorption from soil particles and external transport processes; consequently, the observed biodegradation rate reflects the coupled effects of contaminant bioavailability, mass transfer, and microbial activity [16].

## 6. Analytical Techniques to Assess PTEs in Bioleaching

When assessing PTEs bioleaching efficiency in bioslurries, it is important to determine PTE concentration in both liquid leachates and residual soil. For this purpose, the availability of suitable analytical methods is of paramount relevance.

The procedures for identifying and quantifying PTEs vary depending on the element’s chemical properties, the matrix type, and the chosen/available analytical technique. While direct analysis of the residual solid phase is possible only using techniques such as laser ablation (LA) coupled with inductively coupled plasma mass spectrometry (ICP-MS), laser-induced breakdown spectroscopy (LIBS), or X-ray fluorescence spectroscopy (XRF), most analyses are conducted on liquids, either on filtered leachate or on digested soil.

While the PTE determination in leachate is quite straightforward, soil samples usually need pretreatment steps, typically including oven drying to remove moisture, homogenization by grinding and, for techniques requiring the sample in the liquid form (e.g., atomic absorption spectroscopies—AAS, ICP-MS, ICP optical emission spectroscopy—ICP-OES, electrochemical methods), dissolution in an appropriate acid mixture to release the metals fully into solution [141]. Filtration is then applied to remove the residual particulate.

The selection of the analytical technique also depends on factors including cost, sample throughput, detection limits, sensitivity, regulatory requirements, and matrix complexity. Instrumental techniques for PTE analyses are broadly classified into spectroscopic and electrochemical methods, each with specific advantages and limitations [141].

Currently, most analytical approaches rely on absorption/emission or mass spectrometric techniques, including XRF, ICP-MS, or ICP-OES, atomic emission spectroscopy (AES), and AAS [142]. For instance, Gómez and Bosecker (1999) [50] employed ICP-MS and XRF for the assessment of metal contents in soil supernatants obtained from bioleaching of soils using inoculated *Thiobacillus ferrooxidans* and *Thiobacillus thiooxidans.* In most cases, bioleaching trials typically depend on the analytical method, such as ICP-OES, ICP-MS, or AAS, used for PTE quantification.

Recently, Peralta et al. [143] successfully employed portable XRF (pXRF) in combination with single leaching tests for the rapid risk assessment of Cu, Pb, Zn, and As. Another emerging type of XRF spectroscopy method is total reflection X-ray fluorescence (TXRF), which provides significantly lower detection limits compared to XRF, typically in the range of 10^−7^ to 10^−12^ g. In addition, TXRF can be applied to both solid and liquid samples, while enabling the simultaneous determination of multiple elements with a single measurement [144]. Despite its advantages, the use of TXRF in bioleaching studies is still limited to research and has never been used as a routine analytical tool within the bioreactor framework. Consequently, the application of TXRF remains largely unexplored. However, according to Marguí et al. [145], TXRF has the potential to be deployed for both screening and qualitative or quantitative analysis of leaching solutions.

Another advanced analytical technique that has the potential to provide rapid analysis, in situ measurement, and minimal sample preparation is LIBS. This technique employs a pulsed laser to ablate and atomize the material, forming a transient plasma that emits characteristic elemental spectral lines, allowing both qualitative and quantitative elemental analysis [146]. The LIBS method can analyze a wide range of materials, including gases, solids, liquids, and aerosols. Although LIBS has become an important analytical tool in many scientific fields, including environmental monitoring, its adoption for assessment of PTEs in soil samples is still limited. For example, Senesi et al. [147] showed that LIBS can reliably analyze Mg, Al, Si, Ca, Ti, V, Cr, Mn, Cu, Zn, and Pb in one sewage sludge and five soil samples. Quantitative analysis based on calibration curves was possible for V, Cr, Cu, Zn, and Pb. Likewise, Ma et al. [148] used LIBS for the direct analysis of PTEs, including Cd. Hence, LIBS has the potential to be considered for the analysis of PTEs in soil leachates or soil residues after bioleaching. Progress in LIBS soil analysis was reviewed by Nicolodelli et al. [149].

Furthermore, Arroyo et al. [150] reported that the PTE concentrations measured by LA-ICP-MS were comparable to data from solution ICP-MS analysis in terms of accuracy, precision, and limits of detection. Later, Shaheen et al. [151] validated the use of LA-ICP-MS for PTE quantification in soil. Additionally, Jantzi and Almirall [152] used both LIBS and LA-ICP-MS for soil elemental analysis. Although LA-ICP-MS has been primarily developed for solid sample analysis, dried-droplet deposition techniques have also allowed the determination of trace metals in liquid samples [153]. Analytical principles, advantages, and limitations of these innovative techniques are listed in Table 9, all of them having the potential for application in PTE monitoring in bioleaching research and technology.

## 7. Conclusions

Despite BSRs having been mostly used for organic pollutant removal from contaminated soils, the possibility to use this technique also for PTE remediation is increasingly made possible by advances in bioslurry reactor technology, microbiology, genetics, real-time monitoring, and bacteria immobilization strategies.

Indeed, bioslurry systems are inherently more effective for organic pollutants because they rely on enhanced desorption and microbial degradation pathways that are well suited for organic compounds such as hydrocarbons and pesticides. In contrast, PTE remediation depends on microbial processes—biosorption, bioaccumulation, bioprecipitation, or bioleaching—that are more difficult to sustain under slurry conditions. Maintaining stable microbial performance is challenging due to PTE toxicity, shifts in redox conditions, and competition among microbial strains. Moreover, the selection of suitable carriers remains a critical bottleneck: carriers must ensure high microbial loading, mechanical stability, and resistance to metal-induced deterioration, yet many available materials show limited durability or inconsistent performance over time. These constraints highlight the need for more robust immobilization strategies and engineered carrier materials to extend bioslurry applications to metal-contaminated matrices.

Thus, the development of new support materials that enable rapid biomass retention with long-term operational stability is essential.

Traditional polymeric and AC carriers present economic and environmental challenges, as many are derived from non-renewable fossil resources, are difficult to degrade, and may generate secondary pollution. Industrial AC is commonly produced from coal, wood, coconut shell, peat, or lignite, with costs ranging from $1000–$1500 per ton, and its production contributes to environmental impacts such as ecotoxicity, ozone depletion, greenhouse gases emissions, smog formation, and waste handling issues. Likewise, plastic-based carriers pose concerns due to high production and transportation costs and environmental impacts including energy consumption, carbon emissions, and long-term waste management issues [154].

Consequently, the use of traditional carriers is often neither economically feasible nor environmentally sustainable, particularly given the actual persisting concerns about climate change, resource depletion, and pollution. Thus, cost-effective, environmentally friendly alternatives that utilize locally available or reusable materials are increasingly prioritized.

Natural and waste-derived materials, which are often biodegradable, low-cost, abundant, and require minimal processing, while supporting microbial adhesion and biofilm formation, have emerged as promising alternatives for immobilizing microorganisms. For instance, natural organic carriers, including agricultural residues and food wastes, promote microbial growth, serve as carbon sources, and are generally safe for cells, though they can suffer from low mechanical strength and limited reusability. On the other side, natural inorganic carriers, such as zeolites and clay minerals, offer mechanical strength and chemical stability, and their rough surfaces protect microorganisms under variable conditions, although issues such as poor permeability, high flow resistance, and pore clogging remain.

Among innovative carriers that can be used to immobilize cells and enzymes, hybrid and composite materials could provide suitable properties to enhance bioleaching processes. Such materials combine distinct components to generate properties that are not present in individual materials, thus offering a conducive environment for biomolecules and enhancing microbial stability, reusability, and resistance to harsh environmental conditions [155].

Organic–organic hybrids, formed by combining synthetic polymers (e.g., polyalanine, polyacrylonitrile) with biopolymers (e.g., chitosan, cellulose, alginate, lignin), can yield durable, reusable bioleaching systems by leveraging mechanical stability and biocompatibility. Organic–inorganic hybrids integrate organic polymers with inorganic materials like metal oxides, silica, or carbon compounds, supporting microbial adsorption, covalent binding, and encapsulation due to their high strength and affinity. Inorganic–inorganic hybrids, composed solely of inorganic materials, offer high thermal and chemical stability, mechanical resilience, and functional surface groups, thus enhancing biomolecule binding and hydrophilicity.

Scaling up to pilot or industrial scales is another crucial issue in the implementation of BSRs, as it poses challenges in maintaining parameters like aeration, oxygen transfer, and stirring, while preserving process efficiency and productivity. In this context, the use of immobilized microorganisms may allow for high biomass concentrations, efficient solid–liquid separation, and flexible operational control, thus providing advantages over suspended systems such as reduced hydraulic residence time, enhanced resilience, and improved remediation efficiency.

Real-time monitoring of optimal process conditions is essential to improving the efficiency of BSRs. Robust sensors measuring online operational parameters, such as pH, temperature, pressure, feed rates, oxygen levels, and pollutant concentrations, are needed, thus enabling reproducibility, efficiency, and early problem detection. Biosensors offer high selectivity, low detection limits, and reduced sample preparation requirements, which are suitable for determining both essential (e.g., Ca, Fe, Mg, K) and toxic elements (e.g., Hg, Cd, Pb). Biosensors also facilitate multielement analysis and speciation studies, which can be often enhanced through chemometric tools for differentiating analyte responses. In addition, innovative analytical techniques such as XRF, TXRF, LIBS, and LA-ICP-MS could offer advantages to assess final PTE remediation, allowing the direct analysis of soil, without the need of sample dissolution.

From an economic perspective, bioleaching includes advantages such as relatively low chemical and energy requirements as compared to conventional chemical leaching, but its relatively slow kinetics and the necessity for prolonged treatment may limit its economic competitiveness, specifically for large volumes of soil. Hence, BSRs are likely to be most economically attractive for highly contaminated, relatively fine-grained soils or sediments where excavation is feasible and where a substantial fraction of the target PTE is bioavailable or readily leachable and recoverable. Usually, a site-specific techno-economic assessment is necessary before full-scale implementation.

Bio-based technologies, including the use of siderophores, peptides, and bioelectrochemical systems (BESs), are increasingly applied for selective metal recovery from complex effluents [156].

## 8. Future Perspectives

Despite decades of research, bacteria immobilization in BSRs remains largely empirical, with outcomes often optimized retrospectively rather than predicted. Critical considerations should include the support material properties, immobilization method, and bacteria characteristics, such as structure, surface charge, and stability. Rational design approaches that account for these variables, rather than random protocols, are essential to improve microbial activity, stability, and overall performance.

Future research should also focus on understanding the interplay between microorganisms and carriers, as carriers influence microbial storage and efficiency. Functional carriers should offer multilayer protection and suitable triggering mechanisms, while immobilization strategies must be optimized for each carrier type to maximize microbial retention and performance. Emphasis on cost-effective and eco-friendly carriers, including recycled materials and agricultural or industrial wastes, would reduce costs and environmental impact.

Genetic engineering should be increasingly applied to improve the efficiency of bioslurry remediation of soils contaminated with PTEs. Through this approach, genes in microbial cells should be modified to enhance their ability to mobilize, stabilize, or accumulate metals through heterologous gene expression. Indeed, genetically engineered microbes can express functional genes, such as the CZC (cation zinc cadmium) cluster for Cd (component of Cd efflux system), to survive high metal-contaminated environments and facilitate PTE transformation or accumulation.

Furthermore, integrating biosensors inside bioreactors may provide cost-effective alternatives to off-line analytical techniques for detecting PTEs, converting biochemical processes into measurable optical, electrical, or thermal signals.

Nowadays, advances in artificial intelligence (AI) may allow predictive process optimization, rapid adjustments, and workflow improvements by analyzing large datasets, improving productivity, and identifying critical factors in bioreactor performance.

In general, research on soil bioremediation using bioreactors should not only focus on soil/sediment reuse and wastewater characterization, but also on PTE recovery from the leachate, as resource recovery enhances the economic viability of bioprocesses, especially if elements with an economic value are extracted.

Advances in peptide engineering, recombinant systems, and computational approaches have improved metal-binding specificity and tolerance to high PTE concentrations. The integration of AI and BESs with hydrometallurgical techniques may further promote industrial-scale, environmentally sustainable PTE recovery.

Overall, all these approaches demonstrate the potential for implementing bioremediation by combining resource recovery to treat polluted soils more efficiently and sustainably.

## Figures and Tables

**Table 1:** Soil remediation techniques and their applicability, main mechanisms, advantages, limitations, and current uses.

Remediation Technique	Applicability	Main Mechanisms	Advantages	Limitations	Current Uses	Ref.
Surface capping	In situ; suitable for sites with high contamination	Works by physically isolating contaminated soil to prevent migration of pollutants	Simple design; inexpensive; rapid to deploy; strong containment	Applicable only in restricted or localized areas; reduces agricultural or ecological land functions	Commonly employed for contaminated land management	[3,26]
Encapsulation	In situ; appropriate for severe contamination	Combines containment and isolation through engineered barriers	Provides strong containment and is relatively quick to construct	Effective mainly for shallow contamination; limits land use and ecosystem recovery	Frequently applied for radioactive and composite waste remediation	[3,26]
Electrokinetic remediation	In situ; most effective in fine-grained soils with moderate to high contamination	Utilizes electric fields to mobilize and extract contaminants	Removes pollutants on site and minimizes soil disturbance	Technically demanding; slow; less efficient in low-permeability soils	Limited. Under active pilot-scale testing and optimization	[27,28]
Soil flushing	In situ; best suited for coarse soils with moderate-to-high contamination	Employs chemical or surfactant solutions to desorb and transport contaminants	Low cost; easy to operate; involves minimal mechanical disruption	Efficiency declines in clayey soils; potential for groundwater contamination if uncontrolled	Used selectively, often for mixed contamination	[29]
Immobilization/Stabilization	In situ; typically for high contamination	Reduces contaminant mobility by inducing chemical or physical transformations	Economical; rapid implementation; capable of immediate reduction in toxicity	Provides temporary fixation; contaminants may persist; long-term stability uncertain	Temporary approach; not always standardized for full-scale remediation	[3,26,30]
Phytoremediation	In situ; low-to-moderate contamination	Relies on plant uptake, accumulation, or stabilization of pollutants	Aesthetic; low-cost; environmentally friendly; suitable for large-scale applications	Restricted to surface contamination; plant- and metal-specific; slow process	Under continuous evaluation in pilot and field-scale applications	[31]
Bioremediation	In situ; low-to-moderate contamination	Employs microbial metabolism to transform or immobilize pollutants	Sustainable; cost-effective; and non-destructive of soil structure	Ineffective for heavily polluted soils; biological activity may be slow	Applied mainly as a complementary or supportive method	[32]
Bioreactors	Ex situ; wide range of contaminants and concentrations	Employs microbial metabolism to transform, mobilize, or immobilize pollutants	Full control over reaction parameters; reduced risks of environmental contamination	Costly; need of soil excavation and transportation	Mostly used for organic pollutants with limited applications for PTEs	[33]
Vitrification	In situ or ex situ; high contamination	Involves melting contaminated soil to encapsulate pollutants within glass-like matrices	Produces stable, inert material with high containment performance	Extremely energy-intensive; applicable to limited soil volumes; alters soil function	Routinely practiced for highly toxic or hazardous sites	[3,34]
Solidification	In situ or ex situ; high contamination	Converts contaminated soil into solid, less permeable masses using binders	Quick implementation and strong containment	Expensive; results in loss of soil fertility and natural characteristics	Commonly adopted for PTE immobilization	[30]
Landfilling	Ex situ; high contamination	Physically isolates waste through engineered containment systems	Offers immediate risk elimination and high security	High operational cost; requires dedicated land area; non-sustainable	Widely used for final disposal of hazardous wastes	[3]
Soil washing	Ex situ; moderate-to-high contamination	Separates pollutants via mechanical and chemical extraction	Rapid and effective removal of contaminants from soil particles	Causes major soil disruption and texture modification	Regularly implemented for ex situ cleanup operations	[3]

**Table 2:** Main operational parameters of BSRs for soil remediation.

Operational Parameter	Typical Conditions	Function/Significance	Ref.
Soil slurry density	10–30% *w*/*v*	Affects slurry viscosity, mixing, oxygen transfer, and reactor capacity	[15,40]
Soil:water ratio	From 1:5 to 1:25	Determines slurry fluidity and soil–liquid contact	[15]
Particle size	<1 mm	Smaller particles increase surface area and microorganism–solid contact, whereas excessively fine particles can induce settling/handling problems	[15,40]
Initial pH	6.75–7.25; acidophilic bioleaching necessitates substantially lower pH	Controls microbial activity and metal solubility	[15,40,41]
Temperature	25–30 °C	Affects microbial growth and reaction kinetics	[15]
Stirring	200–400 rpm; bioleaching reactor speed is often reactor-specific	Maintains homogeneous suspension and ameliorates mass transfer	[14,15,40]
Aeration	60 L h^−1^	Provides O_2_ and enhances gas–liquid transfer	[14,15]
Dissolved oxygen	90% saturation	Maintains aerobic conditions	[15]
Nutrients	NH_4_Cl, KH_2_PO_4_, and Na_2_HPO_4_; other elements may depend on the bioleaching process	Supplies N, P, and other useful elements for microbial growth and biochemical reactions	[15,41,42]
Inoculum	System-dependent	Provides active microbial population	[15,41]
Eh	Depends on the types of reaction and bacteria	Indicates oxidation–reduction state and drives Fe cycling	[41,42]
Treatment time	Highly variable: from 120 h to 105 days	Determines contact time and overall contaminant removal	[15]
Reactor configuration	Batch, semi-continuous, and continuous	Determines residence time and process control	[14,15]
Reactor scale	Laboratory/intermediate stirred reactors; commercial stirred tanks > 1000 m^3^	Crucial for scale-up and hydrodynamic design	[40]

**Table 3:** Classification of some PTEs according to their biochemical roles in microorganisms (modified from [61,62]).

PTEs	Classification	Biochemical Role
Zn	Essential element	Present in alcohol dehydrogenase, alkaline phosphatase, aldolase, RNA, and DNA polymerase.
Cu	Essential element	Present in cytochrome oxidase, nitrite reductase of denitrifying bacteria, and oxygenases.
Ni	Essential element	Present in urease, hydrogenase, and factor F430
Co	Essential element	Present in coenzyme B12-containing enzymes (glutamate mutase, methylmalonyl-CoA mutase).
Mo	Essential element	Present in nitrate reductase, nitrogenase, xanthine dehydrogenase, and formate dehydrogenase.
Hg, Cd, Pb, As, V, Sn, Tl	Non-essential element	No known biochemical role

**Table 4:** Structure of siderophores synthesized by Gram-positive and Gram-negative bacteria (modified from [70,72,73].

Siderophore Type	Main Binding Group	Example	Chemical Structure
Hydroxamic acids	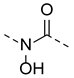 Hydroxamic acid	Desferrioxamine B	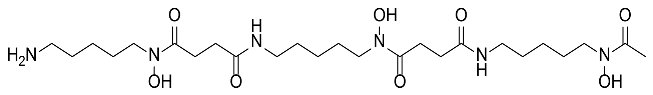
Poly-catechol	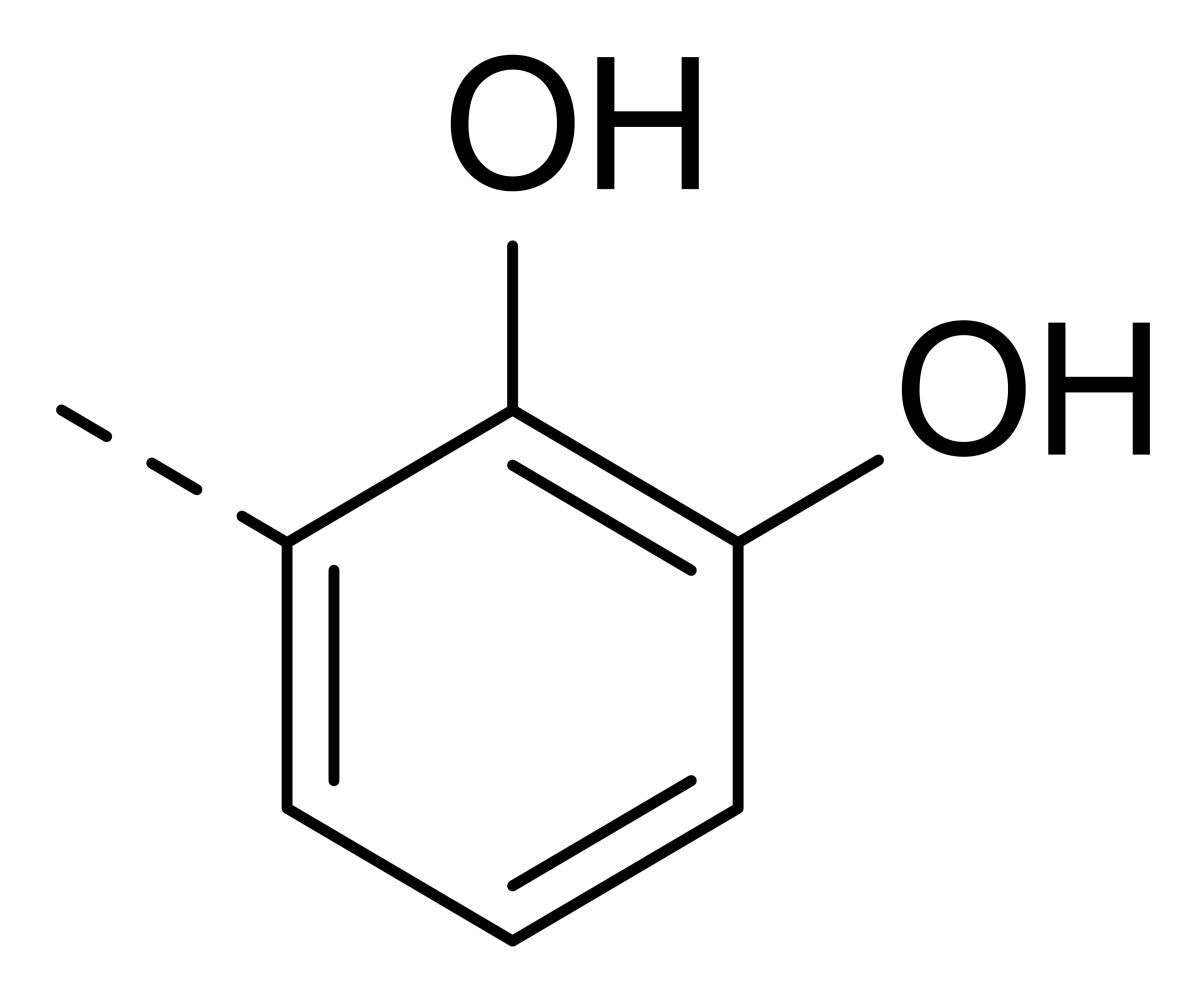 Catechol	Enterobactin	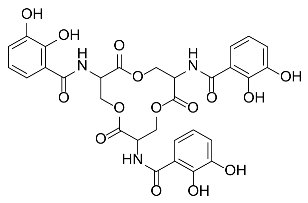
		Salmochelin(s)	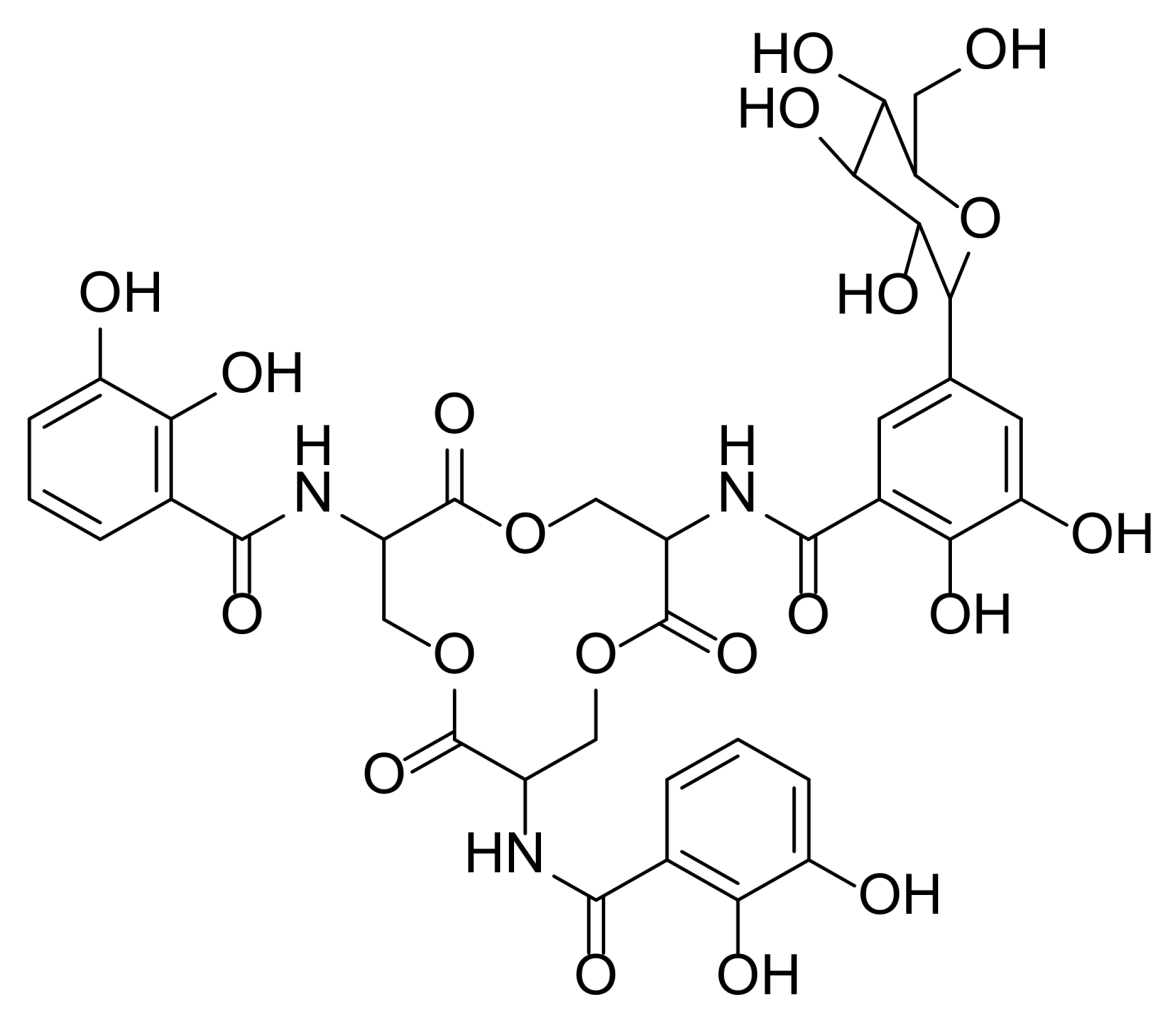
β-hydroxy-carboxyl	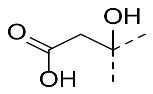 β-hydroxy-carboxyl	Staphyloferrin A	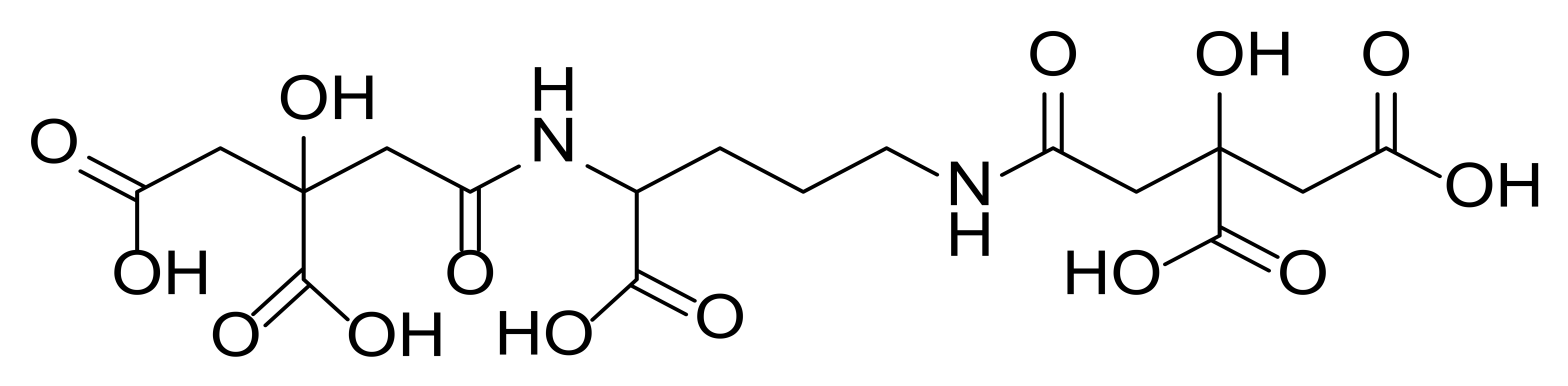
Mixed	Carboxyl Phenolic bi-isothiazole core mono-isothiazole core	Yersiniabactin	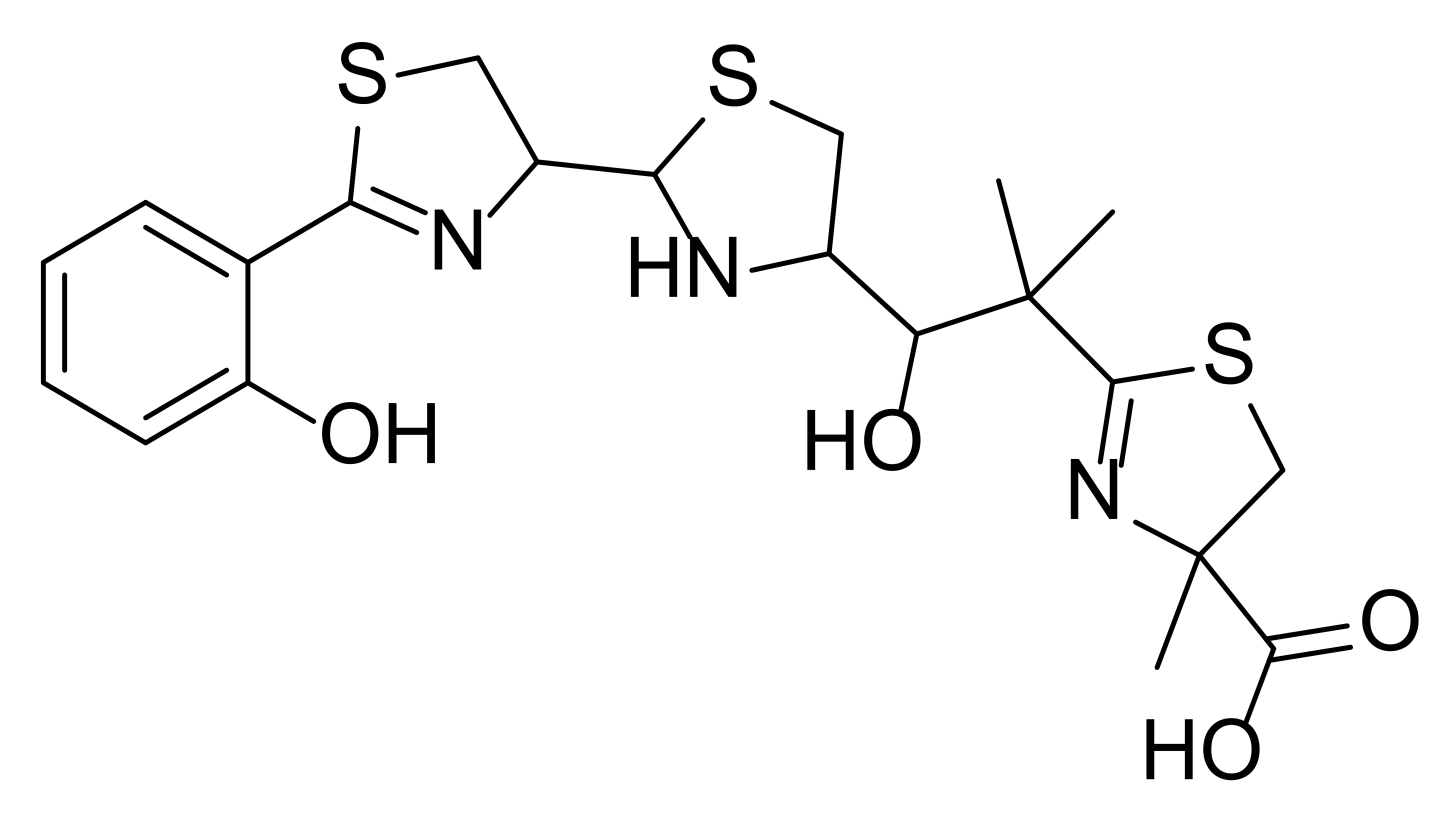
	B-hydroxy-carboxyl Phenolic N-substituted bi-isothiazole core	Pyochelin	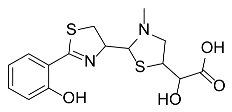
	Hydroxamic acid	Aerobactin	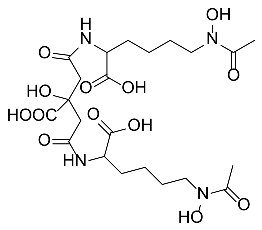
Peptidomimetic	Amide B-hydroxy-carboxylic Pyrimido-isoquinoline diole	Pyoverdin	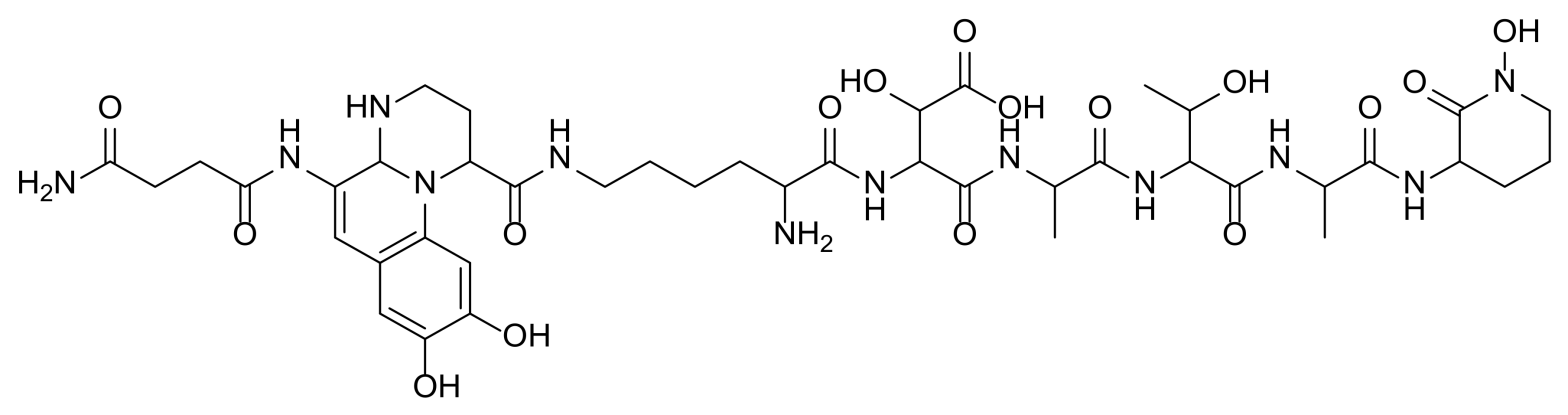

**Table 5:** Microorganisms used in bioleaching.

Microorganism	Metabolism	Optimum Temperature (°C)	Optimum pH	Iron-Oxidizing	Sulfur-Oxidizing	Target PTE	Ref.
*Acidiferrobacter thiooxidans*	Autotroph	≈38	≈2	Yes	No	Cu, Zn, Pb	[90]
*Acidimicrobium ferrooxidans*	Facultative autotroph/mixotroph	45–50	≈2.0	Yes	Yes	Cu, Zn, Ni, Co	[91]
*Acidiphilium cryptum*	Facultative heterotroph	35–40	≈3.5–4.0	No	No	Zn, Pb, Cu	[92]
*Acidithiobacillus albertensis*	Autotroph	25–30	3.5–4.0	Yes	No	Zn, Cr, Cu	[93]
*Acidithiobacillus caldus*	Autotroph	≈45	2.0–2.5	Yes	Yes	Cu, Zn, Ni, Co	[94]
*Leptospirillum ferrooxidans*	Autotroph	28–30	1.5–3.0	Yes	No	Co, Zn, Pb, Cu	[95]
*Pseudomonas putida*	Heterotroph	≈30	7.0–8.0	No	No	Cu, Zn, Pb, Ni	[96]
*Acidianus brierleyi*	Mixotroph	45–75	1.5–2.0	Yes	Yes	Cu	[97]
*Acidianus infernus*	Autotroph	≈90	≈2.0	Yes	Yes	Cu	[98]
*Metallosphaera sedula*	Facultative autotroph/mixotroph	≈75	2.0–3.0	Yes	Yes	Cu	[97]
*Sulfolobus solfataricus*	Mixotroph	≈85	3.0–4.5	No	No	Cu	[99]
*Aspergillus niger*	Heterotroph	35–37	1.5–9.8	No	No	Cu, Zn, Pb, Ni, Cd, Cr	[100]
*Penicillium chrysogenum*	Heterotroph	35–37	3.5–5.5	No	No	Cu, Zn, Pb, Ni	[101]

**Table 6:** Metabolites, substances, and microorganisms used in PTE bioleaching (adapted from [41]).

Metabolite/Microbe/Compound	Role in Bioleaching	Notes/Details
Oxalate	Complexolysis or ligand-induced metal solubilization	Forms complexes with Al, Fe, Mg; facilitates PTE release from mineral lattice; lowers pH
Citrate	Complexolysis	Forms complexes with Ca, Mg; stabilizes PTE ions in solution, enhances leaching; lowers pH
Sulfuric acid (H_2_SO_4_)	Direct and indirect autotrophic bioleaching	Produced via S oxidation; reduces pH and enhances PTE solubilization
Fe^2+^/Fe^3+^	Electron transfer/Oxidation–reduction	Fe^2+^ is oxidized to Fe^3+^ by acidophiles; acts as an electron carrier to facilitate metal sulfide dissolution
Yeast extract/Glucose	Carbon source for heterotrophic bacteria	Provides energy for microbial growth during heterotrophic bioleaching
Biosurfactants	PTE solubilization via micelle formation, ion exchange, precipitation–dissolution, and complexation	Hydrophilic–hydrophobic properties enable extraction of PTEs from soil; micelles trap PTEs
H^+^ (Protons)	Acidolysis	Protonation of oxygen atoms on insoluble PTE compounds; lowers pH
Humic materials/similar compounds	Facilitate microbial Fe(III) and Mn(IV) reduction	Enhances extraction of PTEs from soil
*Acidithiobacillus ferrooxidans*	Acidolysis & electron transfer	Oxidizes Fe^2+^ to Fe^3+^ and reduces sulfur species for metal solubilization
*Leptospirillum ferrooxidans*	Acidolysis	Produces H^+^ via S and Fe oxidation to solubilize PTEs
*Chromobacterium violaceum*	Complexolysis	Produces organic acids and chelators to mobilize PTEs
*Pseudomonas aeruginosa*	Complexolysis	Forms PTE–ligand complexes and solubilizes PTEs via acidolysis/chelation
*Pseudomonas fluorescens*	Complexolysis	Similar function as *P. aeruginosa*; mobilizes PTEs
*Bacillus megaterium*	Complexolysis	Produces chelators and acids to extract PTEs
*Shewanella* spp.	Fe(III)/Mn(IV) reduction	Dissimilatory reduction of Fe(III) and Mn(IV) to Fe(II) and Mn(II), enhancing PTE mobilization
*Geobacter*/*Geobacter metallireducens*	Fe(III)/Mn(IV) reduction; extracellular electron transfer	Reduces insoluble metal oxides; transfers electrons to Fe(III) oxides
*Firmicutes*, *Proteobacteria*, *Deferribacteres*, *Thermotogae*, *Actinobacteria*	Dissimilatory Fe(III) or Mn(IV) reduction	Reduces metals under anaerobic conditions

**Table 7:** Criteria for the selection of support materials for cell and enzyme immobilization (compiled based on [133]).

Selection Criteria	Description/Requirement	Examples of Support Materials
Costs and availability	Should be produced in large quantities and economically feasible	Wood chips, cotton cloth, diatomaceous earth
Mechanical, chemical, and thermal stability	Must withstand operational and storage conditions without degrading	Stainless steel, porous ceramics, epoxy resin
Large surface area	Supports should expose high surface area for cell and substrate interaction	Porous glass, DEAE cellulose, alginate beads
Functional groups for binding	Presence of reactive groups enhances cell adhesion or covalent attachment	Chitosan, polyacrylamide, κ-carrageenan
Maintenance of cell activity	Should not induce lysis or metabolic inhibition	Alginate, gelatin, agar
Ease of handling and disposal	Supports must allow easy recycling or safe disposal	Pectin, PVA
Low contaminant affinity	Minimize non-specific binding to avoid contamination risks	Silica sol, porous ceramics
Optional features	Porosity, swelling, compressibility, biodegradability, microbial growth potential	Natural polymers (agarose, alginate), synthetic hydrogels

**Table 8:** Types of cell immobilization (modified from [136]).

Immobilization Technique	Advantages	Disadvantages
Physical adsorption	Economical and straightforward method; maintains good viability and catalytic performance without requiring chemical reagents.	Weak binding between support and biomolecule may lead to desorption and instability during use.
Entrapment	Permits diffusion of small molecules and ensures long-term operation by retaining cell density within the matrix.	Limited cell loading capacity and potential leakage of cells or enzymes.
Cross-linking	Provides strong interconnections that allow controlled release of reaction products and reduce enzyme or cell loss.	May restrict diffusion of substrates and products, lowering reaction efficiency.
Covalent bonding	Creates durable and heat-resistant linkages ensuring strong attachment of biocatalyst and cells to support.	Often less suitable for whole-cell immobilization due to possible damage to cellular structures.

**Table 9:** Innovative spectroscopic techniques for elemental analysis of soil leachates and residues.

Technique	Analytical Principle	Sample Matrices	Key Advantages	Main Limitations	Relevance for Bioleaching Studies
X-ray Fluorescence (XRF/pXRF)	Measurement of characteristic X-ray emission from elements excited by a primary X-ray source	Mainly solid samples such as soils and sediments	Rapid and non-destructive; minimal sample preparation; portable instruments allow in situ analyses	Relatively high detection limits for trace elements; limited sensitivity for dilute liquid samples	Suitable for rapid screening of PTE contamination in soils before or after bioleaching
Total Reflection X-ray Fluorescence (TXRF)	Reduced background scattering and enhanced sensitivity compared to conventional XRF	Solid and liquid samples, including small-volume solutions	Very low detection limits (10^−7^–10^−12^ g) and multi-element capability	Limited routine use in hydrometallurgical or bioleaching systems	Promising technique for bioleachate analysis due to high sensitivity and small sample volumes
Laser-Induced Breakdown Spectroscopy (LIBS)	Laser pulse generates a micro-plasma whose emission spectrum provides elemental information	Gas, liquid, solid, and aerosol samples	Rapid analysis; minimal sample preparation; enables in situ and multi-element detection	Quantification may be influenced by matrix effects; limited studies on bioleaching leachates and residues	Potential for rapid determination of PTEs in bioleachates and bioleached soil residues
Laser Ablation ICP-MS (LA-ICP-MS)	Laser ablation followed by plasma ionization and mass spectrometric detection of elements	Primarily solid samples; liquids possible via dried-droplet deposition	High sensitivity and precision, and low detection limits	Instrumentally complex; mainly optimized for solid matrices	Potential method for trace PTE determination in bioleached soils and adapted analysis of leachate residues

## Data Availability

No new data were created or analyzed in this study. Data sharing is not applicable to this article.
